# Variations in trajectories of emotional and behavioural symptoms in children and young people with pre‐existing mental health and neurodevelopmental conditions before and during the COVID‐19 pandemic: A nested data linkage clinical cohort study

**DOI:** 10.1002/jcv2.70115

**Published:** 2026-03-17

**Authors:** Brian C. F. Ching, Alice Wickersham, Dominic Stringer, Sarjhana Ragunathan Brindha, Craig Colling, Ruby Morton, Valeria Parlatini, Johnny Downs, Emily Simonoff

**Affiliations:** ^1^ CAMHS Digital Lab Department of Child and Adolescent Psychiatry King's Maudsley Partnership Institute of Psychiatry, Psychology & Neuroscience King's College London London UK; ^2^ Division of Psychiatry University College London London UK; ^3^ Department of Biostatistics and Health Informatics Institute of Psychiatry, Psychology & Neuroscience King's College London London UK; ^4^ Department of Psychological Medicine Institute of Psychiatry, Psychology & Neuroscience King's College London London UK; ^5^ Department of Child and Adolescent Psychiatry Institute of Psychiatry, Psychology & Neuroscience King's College London London UK; ^6^ Centre for Innovation in Mental Health School of Psychology University of Southampton Southampton UK; ^7^ National Institute for Health Research (NIHR) Biomedical Research Centre South London and Maudsley NHS Foundation Trust London UK

**Keywords:** cohort study, COVID‐19 pandemic, data linkage, electronic healthcare records, trajectories

## Abstract

**Background:**

Children and young people with pre‐existing mental health and neurodevelopmental conditions may have experienced heterogeneous mental health impacts during the COVID‐19 pandemic, but what facts may explain these variations are still unclear. We aimed to examine variations in the longitudinal trajectories of emotional and behavioural symptoms before and during the COVID‐19 pandemic in the United Kingdom (UK).

**Methods:**

We used a novel nested clinical cohort study, linking the Maudsley Child and Young People Health and Experience Research (CYPHER) survey and electronic health records (EHRs) data of mental health service users (aged 5–17 years old) in South London and Maudsley NHS Foundation Trust, UK, on 1 June 2020 (*n* = 388). Composite emotional and behavioural scores, including internalising and externalising symptoms, were assessed in June–September 2020 and February–March 2021 by the Maudsley CYPHER survey and the Strengths and Difficulties Questionnaire from EHRs was used to supplement data pre‐pandemic (2019–March 2020) and during the pandemic (2020 and 2021). Sociodemographic characteristics and diagnoses were extracted from EHRs. Relative symptom trajectories were modelled with predictors using linear mixed models.

**Results:**

Relative emotional and behavioural symptoms were not significantly different from pre‐pandemic to 2021, but variations were found across predictors. Those who were female (vs. male; *b* = 0.19, 95% CI 0.02 to 0.36), lived in deprived neighbourhoods (vs. non‐deprived; *b* = 0.19, 95% CI 0.03 to 0.36), and had a diagnosis of autism (vs. emotional disorder; *b* = 0.33, 95% CI 0.13 to 0.53) had relatively higher emotional symptoms pre‐ and during pandemic. Those who were Black (vs. White; *b* = −0.48, 95% CI −0.82 to −0.14) or who did not state their ethnicity (*b* = −0.47, 95% CI −0.82 to −0.12) had relatively higher decreases in emotional symptoms between pre‐pandemic and 2020. Those who had a diagnosis of intellectual disability (vs. without) had greater relative decrease in emotional symptoms between pre‐pandemic and 2020 (*b* = −0.94, 95% CI −1.60 to −0.28) and 2021 (*b* = −2.02, 95% CI −2.46 to −1.58). Those who were younger (vs. older; *b* = −0.03, 95% CI −0.06 to −0.01) and had a diagnosis of autism (vs. emotional disorder; *b* = 0.40, 95% CI 0.20 to 0.60) or attention‐deficit hyperactivity disorder (vs. emotional disorder; *b* = 0.43, 95% CI 0.19 to 0.66) had relatively higher behavioural symptoms pre‐ and during pandemic.

**Conclusion:**

Emotional and behavioural symptoms were high and relatively stable across pre‐ and during pandemic timepoints. Relatively worse emotional and behavioural symptoms were observed in children and young people who were younger, female, lived in deprived neighbourhoods, and had a diagnosis of a neurodevelopmental condition. However, some effects might reflect distinctive features between groups rather than pandemic‐specific differences. Future research should examine longer‐term mental health impacts of the pandemic across clinical groups and potential mechanisms of change.

## Key Points

**What's known?**

- The COVID‐19 pandemic had a varied longitudinal impact on the mental health of children and young people with pre‐existing mental health and neurodevelopmental conditions, but predictors of these variations are unclear.

**What's new?**

- Relative emotional and behavioural symptoms were not significantly different from pre‐pandemic to 2021 with variations present.
- Age, sex, neighbourhood deprivation, ethnicity, and diagnosis may explain variations in mental health trajectories before and during the pandemic.

**What's relevant?**

- These findings emphasise the high clinical needs of children and young people with pre‐existing conditions and highlights potential vulnerable subgroups. Clinical services and policy should consider targeted support for those who may have worse longitudinal outcomes.
- Future research is needed to investigate longer‐term outcomes and mechanisms of change to improve our understanding of the pandemic's mental health impact and inform preparedness to future crises.

## INTRODUCTION

The COVID‐19 pandemic disrupted key developmental milestones in children and young people which may have had consequences for their mental health (Sonuga‐Barke, 2021). Systematic reviews of the general population have suggested some evidence for negative impacts of the pandemic on children and young people's mental health, but findings were largely inconclusive and mixed (Ahmed et al., 2023; Newlove‐Delgado et al., 2023; Robinson et al., 2022; Sun et al., 2023). However, reviews consistently reported that those with pre‐existing mental health and neurodevelopmental conditions fared worse compared to their healthy peers.

A systematic review and meta‐analysis of longitudinal studies on clinical samples found that the pandemic had a varied impact on children and young people with pre‐existing conditions (Ching et al., 2025). Although their meta‐analysis found overall no significant change in emotional and behavioural symptoms, narrative synthesis of individual study findings revealed increases and decreases across subgroups indicating divergent changes in mental health during the pandemic. Other longitudinal studies also found that there were no significant differences in mental health outcomes across different pandemic timepoints (Joensen et al., 2022; Sayal et al., 2023). The Co‐SPACE study found that children and young people with pre‐existing mental health and neurodevelopmental conditions were more likely to see worsened, yet stable emotional and behavioural symptoms during the pandemic compared to peers without pre‐existing conditions (Guzman Holst et al., 2023).

The potential factors that may drive differences in mental health outcomes in clinical groups of children and young people are still unclear. Ching et al.'s (2025) narrative synthesis indicated that factors, such as age, sex, and diagnosis, may explain some of the divergent findings of the pandemic's mental health impact in this group, but associations were inconsistent across studies. Another systematic review examined longitudinal studies that investigated ethnicity and socioeconomic position indicators' association with mental health outcomes of children and young people with pre‐existing conditions during the pandemic (Lee et al., 2026). They found some evidence for worse emotional symptoms in clinical groups from lower income brackets and who experienced financial hardship, but weaker evidence for ethnicity's effect. This paucity of studies on predictors of mental health changes during the pandemic limits our understanding of the reasons for the heterogeneity found in longitudinal mental health outcomes during the pandemic.

Although 5 years from the start of the pandemic, there is still limited understanding of the longitudinal impact of the pandemic on mental health symptoms in children and young people with pre‐existing conditions and what factors may predict different outcomes. Most studies pool effects of heterogeneous clinical groups without examining subgroup differences across sociodemographic and clinical characteristics, which may miss important variations beyond the sample mean (Ching et al., 2025). Due to the urgent nature of the pandemic and the need for rapid data collection, many studies do not report or control for key sociodemographic and clinical factors which may bias estimates. Moreover, most published pandemic studies on clinical groups do not have comprehensive mental health data across pre‐ and during pandemic timepoints, limiting our understanding of the longitudinal changes as a result of the pandemic and related events from 2020 onwards. These crucial learning points may benefit future pandemic responses and improve planning of service provision for children and young people's mental health.

To our knowledge, there has not yet been research that has examined predictors of the variations in mental health trajectories during the pandemic across clinical groups. Thus, we aim to address the limitations of the current literature and examine predictors of trajectories of emotional and behavioural symptoms before and during the pandemic in children and young people with pre‐existing conditions.

## METHODS

This study followed the RECORD reporting guidelines (Benchimol et al., 2015; Appendix S1).

### Design and sample

We used a novel nested clinical cohort study linking survey and electronic health records (EHR) data where survey data for a defined cohort was collected and ingested into the wider EHR sample. The South London and Maudsley NHS Foundation Trust Biomedical Research Centre (SLaM BRC) Case Register contains pseudonymised EHRs from secondary mental health services in South London, UK, including mental health services for children and young people. We sampled 5386 children and young people (aged 5–17) who were active outpatients in community and specialist services for children and young people on 1 June 2020.

Caregivers and children and young people (with parental consent if below 16) were invited to complete the Maudsley Child and Young People Health and Experience Research (CYPHER) survey (Parlatini et al., 2024) across three pandemic waves (June–September 2020, February–March 2021, and March–May 2022) as part of patient monitoring activities under the UK legal framework of Regulation 3(2)/3(3) of the Health Service Control of Patient Information 2002 (COPI). The survey aimed to investigate the mental health impact of the pandemic on children and young people attending mental health services in South London, based on the CoRonavIruS Health Impact Survey (CRISIS) survey (Nikolaidis et al., 2021), including emotional and behavioural symptoms, caregiver mental health, and pandemic‐related factors. Children and young people who were accessing inpatient services were excluded as they represent a minority with specific needs and severe presentations.

Survey data were then nested within their EHRs. This linkage was conducted at the individual level using anonymised identifiers (patient numbers) and the Clinical Record Interactive Search (CRIS) data resource, a tool which allows access to datasets derived from mental health service EHRs in South London (Stewart et al., 2009). Pre‐pandemic sociodemographic characteristics (age, sex, ethnicity, and neighbourhood deprivation) and clinical data (diagnoses and routine collected patient reported outcome measures) were extracted from CRIS and matched to the available individual survey responses.

In this paper, we only present caregiver‐reported data from the first two waves. The number of cases with pre‐pandemic and 2020–2022 data and both caregiver and self‐reported data in the Maudsley CYPHER survey and EHRs was too small and does not reflect the range of children and young people receiving care at the beginning of the survey, most of whom would have been discharged from services by 2022. Thus, only cases with data in pre‐pandemic and 2020–2021 were included in the analytic sample. CRIS has research ethics approval for secondary analyses from South Central—Oxford C Research Ethics Committee (reference 23/SC/0257), and this specific project was approved by the SLaM CRIS oversight committee (number 23‐021).

### Outcomes

Emotional and behavioural symptom trajectories were estimated using two measures of caregiver‐reported emotional and behavioural symptoms. In this study, we use the term trajectories to describe longitudinal patterns of emotional and behavioural symptoms across pre‐pandemic, 2020, and 2021 timepoints at the group level. In the Maudsley CYPHER survey, emotional and behavioural symptoms were assessed at waves 1–2 using measures adapted from the CRISIS survey. Separate emotional and behavioural composite scores were created and validated based on results from factor analysis (Parlatini et al., 2024; Appendix S2). Likert scores of individual symptoms were summed to generate a caregiver‐reported total emotional (range 6–30) and behavioural score (range 5–20), with higher scores indicating more severe symptoms.

However, pre‐pandemic emotional and behavioural symptoms were not captured by the Maudsley CYPHER survey, so caregiver‐reported Strengths and Difficulties Questionnaire (SDQ) extracted from EHRs were used to supplement data. Pre‐pandemic, all SDQs were extracted between January 2019 and March 2020 with the earliest SDQ per patient selected. SDQ data was also used to supplement the Maudsley CYPHER survey emotional and behavioural scores to maximise data points during the pandemic; if a patient did not respond to the Maudsley CYPHER survey but had SDQ data in their EHR, they could be included. The earliest SDQ data in 2020 (April–December 2020) and 2021 (January–December 2021) per patient was used to maximise temporal proximity between the Maudsley CYPHER survey and EHR data. Separate emotional and behavioural scores were created by combining emotional with peer subscale, and conduct with hyperactivity subscale scores together, respectively (Goodman et al., 2010). Emotional and behavioural scores range from 0 to 20, where higher scores indicate more symptoms.

To ensure the two emotional and behavioural symptom scores were comparable across measures and timepoints, all raw scores were *z*‐standardised within each timepoint (pre‐pandemic, 2020, and 2021), with the mean as 0 and standard deviation (SD) as 1. This was done separately for emotional and behavioural symptom scores at each timepoint and separately for the SDQ and Maudsley CYPHER survey (Appendix S3). In 2020 and 2021, as both SDQ and the Maudsley CYPHER survey data were available, separate composite emotional and behavioural symptom *z*‐scores were calculated by averaging the corresponding SDQ and Maudsley CYPHER *z*‐scores. The final *z*‐scores represented standardised emotional and behavioural symptom scores that were comparable across measures and timepoints; a 0.1 change in *z*‐score roughly translated to 0.4 and 0.3 change across timepoints for raw emotional and behavioural scores, respectively (Appendix S4).

### Potential predictors

Sociodemographic characteristics include age at start of the pandemic (continuous variable) and sex (binary variable; male, female). Age was also treated as a categorical variable (<12 years old/primary school, 12–16 years old/secondary school, and >16 years old/college age) for ease of interpretation in visualisations. Ethnicity was defined according to the UK Office for National Statistics guidelines and collapsed into the following categories: White, Black, Asian, Mixed, or Other, not stated. Neighbourhood deprivation quintiles were derived from Index of Multiple Deprivation (IMD) scores provided by the Office of National Statistics. We converted the IMD scores into five categories of increasing socio‐economic deprivation and further collapsed it into a binary variable for ease of interpretation: deprived (most and second most deprived) and not deprived (third, second and least deprived). This approach, based on nationally defined cut‐points, was chosen over a sample‐specific median split to ensure representativeness relative to the wider population. Primary diagnosis and intellectual disability (ID) diagnosis recorded by clinicians were based on the International Classification of Diseases 10th edition (ICD‐10; Appendix S5). Primary diagnosis was categorised by the three largest primary diagnostic groups, which included attention‐deficit/hyperactivity disorder (ADHD), autism spectrum disorder (ASD), and emotional disorders (such as depressive and anxiety disorders). In this paper, we use the term ‘diagnosis of ASD/autism’ to reflect the presence of a recorded clinical diagnosis.

### Statistical analysis

Statistical analyses were conducted in Stata 17. Logistic regressions examined whether study predictors were associated with inclusion in the analytic sample (having emotional and behavioural symptoms data in pre‐pandemic and 2020–2021). From these models, each participant's predicted probability of inclusion was obtained, and inverse probability weights were derived as the reciprocal of these probabilities. Descriptives for sample characteristics and mean emotional and behavioural symptom scores across waves and predictors were reported. Separate linear mixed models with random intercepts for individuals were fitted using the Stata ‘mixed’ function, with weights applied, to examine relative changes in emotional and behavioural symptoms between pre‐pandemic, 2020, and 2021. Maximum likelihood estimation was used to estimate both fixed and random effects (Bates et al., 2015). Random slopes for year were tested but did not improve model fit and were therefore not included (Appendix S6). Symptom trajectories were modelled with all predictors (age, sex, ethnicity, neighbourhood deprivation, primary diagnosis, and ID) included in the same model to control for each other's effects. Interactions between time and significant predictors were explored and included in models if significant. As emotional and behavioural symptom scores were standardised, coefficients for time reflect relative differences rather than absolute changes over time. We conducted subgroup analyses investigating potential differences in predictors across primary diagnoses by rerunning the models separately in the ADHD, ASD, and emotional disorders groups. This was informed by previous literature that suggested heterogeneity in potential explanatory factors across diagnostic groups during the pandemic, such as sex and financial hardship (Ching et al., 2025). We also conducted a sensitivity analysis to investigate robustness of results by repeating the models with complete cases without weights applied.

## RESULTS

**FIGURE 1:**
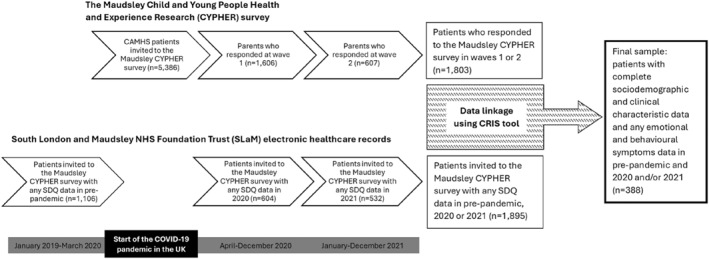
Patient flow diagram of our data linkage clinical cohort study (Maudsley CYPHER survey and SLaM electronic healthcare records).

In total, 5386 children and young people and their caregivers were invited to complete the Maudsley CYPHER survey. At waves 1 and 2, 1606 (29.8%) and 607 (11.3%) caregivers responded, respectively, with 1803 (33.5%) young people whose caregivers responded to the survey in waves 1 and/or 2 (Figure 1). In the EHRs, 1106 (20.5%), 604 (11.2%), and 532 (9.9%) children and young people had any SDQ data in pre‐pandemic, 2020, and 2021, respectively; 1895 (35.2%) had any SDQ data in either pre‐pandemic, 2020, or 2021. The final analytic sample was 388 children and young people with complete sociodemographic and clinical characteristic data and any emotional and behavioural symptoms data in pre‐pandemic and 2020 and/or 2021. See Table 1 for sample characteristics of the analytic sample and Tables S1–S7 for raw emotional and behavioural symptoms scores before *z*‐standardisation across time and by age, sex, ethnicity, neighbourhood deprivation, primary diagnosis, and ID diagnosis.

**TABLE 1:** Sample characteristics of children and young people in the analytic sample.

Median (SD), range	Analytic sample (*n* = 388)
Age	12.32 (3.23), 5.44 to 18.44

*n* (%)	
Sex	
Male	242 (62.37%)
Female	146 (37.63%)
Ethnicity	
White	198 (51.03%)
Black	59 (15.21%)
Asian, mixed, and other	62 (15.98%)
Not stated	69 (17.78%)
Neighbourhood deprivation	
Not deprived	312 (80.41%)
Deprived	76 (19.59%)
Main three diagnosis	
ADHD	84 (21.65%)
ASD	197 (50.77%)
Emotional disorders	107 (27.58%)

*Note*: The analytic sample included young people within the larger CAMHS cohort with complete sociodemographic and clinical characteristic data and any emotional and behavioural symptoms data in pre‐pandemic and 2020 and/or 2021. The intellectual disability variable in the analytic sample has not been reported due to potentially disclosive cell counts in line with statistical disclosure guidance.

Abbreviations: ADHD, attention‐deficit/hyperactivity disorder; ASD, autism spectrum disorder; CAMHS, child and adolescent mental health services; SD, standard deviation.

Logistic regression analysis found that compared to those without sufficient data in the Maudsley CYPHER survey and EHRs, those included in the analytic sample were more likely to be younger, have a primary diagnosis of ASD or emotional disorders, and less likely to be Black or have a diagnosis of ID (Table S8).

### Emotional symptom trajectories

In pre‐pandemic, mean SDQ emotional score was 13.19 (4.14). In 2020 and 2021, mean SDQ emotional score was 12.76 (4.37) and 13.35 (4.45), respectively, and mean Maudsley CYPHER emotional score was 18.08 (4.92) and 17.55 (4.73), respectively. Raw scores were not significantly different across time (Table S2).

Across pre‐ and during pandemic timepoints, females had relatively higher emotional symptoms (small effect of ∼0.2 SD) than males (*b* = 0.19, SE = 0.08, *Z* = 2.24, 95% CI 0.02 to 0.36, *p* = 0.025). Compared to those living in non‐deprived neighbourhoods, children and young people in deprived areas reported relatively higher emotional symptoms (small effect of ∼0.2 SD; *b* = 0.19, SE = 0.09, *Z* = 2.28, 95% CI 0.03 to 0.36, *p* = 0.022). Young people with ASD had relatively higher emotional symptoms (moderate effect of ∼0.3 SD) compared with those with emotional disorders (*b* = 0.33, SE = 0.10, *Z* = 3.24, 95% CI 0.13 to 0.53, *p* = 0.001). No differences were observed by age (Table 2). There was no evidence for interactions between sex, neighbourhood deprivation, and primary diagnosis with time and thus not included in the model (Table S9).

**TABLE 2:** Estimated trajectories of emotional symptoms between pre‐pandemic and 2021 with inverse probability weights.

Predictor	Fixed effect				
	*b*	SE	*Z*	95% CI	*p*
Year (slope)^a^					
Pre‐pandemic	Reference				
2020	0.17	0.11	1.56	−0.04 to 0.37	0.119
2021	0.12	0.15	0.84	−0.17 to 0.41	0.403
Age at start of pandemic	0.02	0.01	1.41	−0.01 to 0.05	0.159
Sex					
Male	Reference				
Female	0.19	0.08	2.24	0.02 to 0.36	0.025
Ethnicity					
White	Reference				
Black	0.18	0.15	1.24	−0.10 to 0.46	0.216
Asian, mixed, or other	0.01	0.15	0.06	−0.29 to 0.30	0.956
Not stated	−0.05	0.15	−0.33	−0.34 to 0.24	0.742
Neighbourhood deprivation					
Not deprived	Reference				
Deprived	0.19	0.09	2.28	0.03 to 0.36	0.022
Primary diagnosis					
Emotional disorders	Reference				
ADHD	−0.19	0.12	−1.57	−0.42 to 0.05	0.117
ASD	0.33	0.10	3.24	0.13 to 0.53	0.001
Intellectual disability					
No or not stated	Reference				
Yes	0.20	0.20	0.97	−0.20 to 0.60	0.334
Year#Ethnicity					
2020#Black	−0.48	0.17	−2.74	−0.82 to −0.14	0.006
2020#Asian, mixed, and other	−0.20	0.19	−1.17	−0.59 to 0.15	0.242
2020#Not stated	−0.47	0.18	−2.61	−0.82 to −0.12	0.009
2021#Black	−0.32	0.26	−1.21	−0.83 to 0.20	0.227
2021#Asian, mixed, and other	0.13	0.24	0.55	−0.34 to 0.60	0.581
2021#Not stated	−0.13	0.20	−0.64	−0.53 to 0.27	0.522
Year#Intellectual disability					
2020#Yes	−0.94	0.34	−2.79	−1.60 to −0.28	0.005
2021#Yes	−2.02	0.22	−9.02	−2.46 to −1.58	0.000
Intercept (reference groups)	−0.46	0.22	−2.07	−0.89 to −0.03	0.038

	Random effect		
	Estimate	SE	95% CI
Variance (intercept)	0.53	0.04	0.45 to 0.61
Variance (residual)	0.32	0.03	0.27 to 0.38
ICC	0.62	0.03	0.56 to 0.67

*Note*: This table represents data from linear mixed modelling using the analytic sample, which included young people within the larger CAMHS (child and adolescent mental health services) cohort with complete sociodemographic and clinical characteristic data and any emotional and behavioural symptoms data in pre‐pandemic and 2020 and/or 2021; Inverse probability weights were applied to the analysis, which were based on logistic regressions examining whether study predictors were associated with inclusion in the analytic sample (having emotional and behavioural symptoms data in pre‐pandemic and 2020–2021). From these models, each participant's predicted probability of inclusion was obtained, and inverse probability weights were derived as the reciprocal of these probabilities.

Abbreviations: #, interaction term; 95% CI, 95% confidence interval; ADHD, attention‐deficit/hyperactivity disorder; ASD, autism spectrum disorder; *b*, fixed effect coefficient; ICC, intraclass correlation coefficient; ID, intellectual disability; *p*, *p*‐value; SE, standard error; *z*, *z*‐score.

^a^ Outcomes were *z*‐standardised within each timepoint (pre‐pandemic, 2020, 2021); therefore, coefficients for time reflect relative changes in symptoms.

Significant interactions emerged between time and both ethnicity and ID diagnosis. Compared to White ethnic groups, those from Black ethnic groups (*b* = −0.48, SE = 0.17, *Z* = −2.74, 95% CI −0.82 to −0.14, *p* = 0.006) or who did not state their ethnicity (*b* = −0.47, SE = 0.18, *Z* = −2.61, 95% CI −0.82 to −0.12, *p* = 0.009) showed greater relative decrease in emotional symptoms between pre‐pandemic and 2020 (moderate effects of ∼0.5 SD). Compared to those without an ID diagnosis, those with ID showed greater relative decrease in emotional symptoms between pre‐pandemic and both 2020 (*b* = −0.94, SE = 0.34, *Z* = −2.79, 95% CI −1.60 to −0.28, *p* = 0.005) and 2021 (*b* = −2.02, SE = 0.22, *Z* = −9.02, 95% CI −2.46 to −1.58, *p* = 0.001).

### Behavioural symptom trajectories

In pre‐pandemic, mean SDQ behavioural score was 12.66 (4.39). In 2020 and 2021, mean SDQ behavioural score was 12.72 (4.35) and 12.69 (3.90), respectively, and mean Maudsley CYPHER behavioural score was 13.11 (3.78) and 12.96 (3.36), respectively. Raw scores were not significantly different across time (Table S2).

Across pre‐ and during pandemic timepoints, younger service users had relatively higher behavioural symptoms (small effect of ∼0.05 SD) compared to older counterparts (*b* = −0.03, SE = 0.01, *Z* = −2.49, 95% CI −0.06 to −0.01, *p* = 0.013). Children and young people with a primary diagnosis of ADHD (*b* = 0.43, SE = 0.12, *Z* = 3.55, 95% CI 0.19 to 0.66, *p* = 0.000) or ASD (*b* = 0.40, SE = 0.10, *Z* = 3.91, 95% CI 0.20 to 0.60, *p* = 0.000) had relatively higher behavioural symptoms (moderate effects of ∼0.4 SD) than those with emotional disorders. There was no evidence for interactions between age and primary diagnosis with time and thus not included in the model (Table S10). No differences were observed by sex, ethnicity, neighbourhood deprivation, or ID diagnosis (Table 3).

**TABLE 3:** Estimated trajectories of behavioural symptoms between pre‐pandemic and 2021 with inverse probability weights.

Predictor	Fixed effect				
	*b*	SE	*Z*	95% CI	*p*
Year (slope)^a^					
Pre‐pandemic	Reference				
2020	−0.06	0.09	−0.69	−0.23 to 0.11	0.491
2021	−0.11	0.09	−1.20	−0.28 to 0.07	0.229
Age at start of pandemic	−0.03	0.01	−2.49	−0.06 to 0.01	0.013
Sex					
Male	Reference				
Female	−0.01	0.08	−0.09	−0.17 to 0.16	0.926
Ethnicity					
White	Reference				
Black	−0.04	0.10	−0.35	−0.24 to 0.16	0.723
Asian, mixed, or other	0.00	0.11	0.04	−0.21 to 0.22	0.968
Not stated	−0.11	0.11	−1.07	−0.32 to 0.09	0.285
Neighbourhood deprivation					
Not deprived	Reference				
Deprived	0.13	0.10	1.39	−0.05 to 0.32	0.165
Primary diagnosis					
Emotional disorders	Reference				
ADHD	0.43	0.12	3.55	0.19 to 0.66	0.000
ASD	0.40	0.10	3.91	0.20 to 0.60	0.000
Intellectual disability					
No or not stated	Reference				
Yes	0.01	0.18	0.06	−0.34 to 0.36	0.950
Intercept (reference groups)	0.19	0.21	0.91	−0.22 to 0.60	0.365

	Random effect		
	Estimate	SE	95% CI
Variance (intercept)	0.51	0.04	0.44 to 0.59
Variance (residual)	0.38	0.03	0.33 to 0.45
ICC	0.57	0.03	0.51 to 0.63

*Note*: This table represents data from linear mixed modelling using the analytic sample, which included young people within the larger CAMHS (child and adolescent mental health services) cohort with complete sociodemographic and clinical characteristic data and any emotional and behavioural symptoms data in pre‐pandemic and 2020 and/or 2021; Inverse probability weights were applied to the analysis, which were based on logistic regressions examining whether study predictors were associated with inclusion in the analytic sample (having emotional and behavioural symptoms data in pre‐pandemic and 2020–2021). From these models, each participant's predicted probability of inclusion was obtained, and inverse probability weights were derived as the reciprocal of these probabilities.

Abbreviations: #, interaction term; 95% CI, 95% confidence interval; ADHD, attention‐deficit/hyperactivity disorder; ASD, autism spectrum disorder; *b*, fixed effect coefficient; ICC, intraclass correlation coefficient; ID, intellectual disability; *p*, *p*‐value; SE, standard error; *z*, *z*‐score.

^a^ Outcomes were *z*‐standardised within each timepoint (pre‐pandemic, 2020, 2021); therefore, coefficients for time reflect relative changes in symptoms.

### Subgroup analyses

Minor differences were observed between diagnostic groups in predictors of emotional and behavioural symptoms (Tables S11  and S12). For emotional symptoms, effects of sex, ethnicity, and neighbourhood deprivation were no longer maintained in separate diagnostic groups. The effect of ID diagnosis on emotional symptoms could only be examined in the ASD group due to insufficient numbers in other groups, and this association remained. For behavioural symptoms, the effect of age was maintained only in the ASD group. In the ADHD group, an effect of ethnicity was found where children and young people who did not state their ethnicity reported relatively higher behavioural symptoms (moderate effect of ∼0.4 SD) relative to White peers across pre‐ and during pandemic timepoints (*b* = 0.44, SE = 0.18, *Z* = 2.46, 95% CI 0.09 to 0.79, *p* = 0.014).

### Sensitivity analysis

Repeating the main and subgroup analyses without survey weights produced largely consistent results with few differences (Tables S13–S16). For emotional symptoms, compared to White peers, those who did not state their ethnicity no longer showed greater relative decrease in emotional symptoms between pre‐pandemic and 2020 (Table S13). The time‐dependent effect of ID diagnosis on emotional symptoms was also not observed. Within the ASD group, the effect of ID diagnosis was attenuated when weights were removed but an effect of sex on emotional symptoms was found (small effect of ∼0.2 SD; *b* = 0.23, SE = 0.12, *Z* = 2.02, 95% CI 0.01 to 0.46, *p* = 0.043; Table S14).

## DISCUSSION

We used a nested data linkage clinical cohort study combining longitudinal survey and EHR data to examine variations in trajectories of emotional and behavioural symptoms in children and young people with pre‐existing mental health and neurodevelopmental conditions in the UK between before and during the pandemic (2020–2021). We found that emotional symptoms and behavioural symptoms were high and relatively stable across pre‐ and during pandemic timepoints. Existing meta‐analyses and studies with clinical samples of children and young people also found that mental health outcomes did not vary during the pandemic (Ching et al., 2025; Guzman Holst et al., 2023; Joensen et al., 2022; Sayal et al., 2023). This may be because this group, often awaiting or attending services and dealing with multiple stressors, already had high clinical need before the pandemic. We may be observing a ‘ceiling effect’ where high symptoms before the pandemic may not have significantly changed during the pandemic but still pose high morbidity.

Compared to children and young people who were males and lived in non‐deprived neighbourhoods, children and young people who were female and lived in deprived neighbourhoods had ∼0.2 SD higher emotional symptom scores before and during the pandemic. This is consistent with existing literature, where a longitudinal probability sample survey of the UK general population indicated that before the pandemic, females aged 16–24 already had higher proportions of mental health problems compared to males and saw the sharpest increase at the start of the pandemic (Pierce et al., 2020). Similarly, being female was associated with worse depressive and anxiety symptoms in those with pre‐existing conditions, such as ASD, ADHD, and depression, across pre‐ and during the pandemic timepoints (Dvorsky et al., 2022; Sadeghi et al., 2022; Toseeb & Asbury, 2023). Another study found that children and young people from households with incomes below £16k per year were most likely to have higher emotional symptom trajectories during the pandemic (Guzman Holst et al., 2023). Analysis of longitudinal studies also found that children and young people with pre‐existing conditions from lower income brackets were more likely to have increased emotional symptoms compared to those in higher income groups (Lee et al., 2026). This suggests that sex and neighbourhood deprivation may be explanatory factors that consistently impact children and young people's mental health outside and within pandemic contexts.

Age was a significant predictor of behavioural symptom trajectories, where younger children and young people had slightly relatively worse behavioural symptoms over time than adolescents. This aligns with epidemiological data of general populations and clinical surveys of children and young people that consistently find being younger is associated with significantly increased rates of mental health problems, including oppositional defiant and conduct problems (Guzman Holst et al., 2023; Li et al., 2022).

Children and young people with neurodevelopmental conditions overall had relatively worse outcomes during the pandemic than those without, with ∼0.3–0.4 SD higher emotional and behavioural symptom scores. This aligns with previous research that autistic children and young people and those with ADHD and other needs (e.g., special education and learning) may be at higher risk of poorer mental health before (Beesdo et al., 2009; van Steensel et al., 2011) and during the pandemic compared to neurotypical peers (Conti et al., 2020; Guzman Holst et al., 2023; Houghton et al., 2022; Parlatini et al., 2024). The worsening may be due to lower tolerance of uncertainty and loss of routine, school disruptions, lack of support, and increased rates of mental health problems, which were highlighted by qualitative interviews with neurodivergent young people and exacerbated during the pandemic (Asbury & Toseeb, 2023). Additionally, increased behavioural symptoms in neurodivergent groups relative to neurotypical youth may also reflect distinctive core features between diagnostic groups, rather than pandemic‐specific effects. However, emotional symptoms seemed to have decreased within the ASD group. Asbury and Toseeb (2023) found improvements in some autistic youth's mental health during the first 6‐months of the pandemic because of reduced demand and the absence of stressors. Poorer mental health in this group may have been maintained by pre‐existing inequalities and additional pandemic‐related stressors, but variations may exist across diagnostic groups.

Compared to White ethnic groups, children and young people from Black ethnic groups showed greater relative decrease in emotional symptoms in 2020 but not 2021 compared to White counterparts. This corresponded to ∼0.5 SD lower emotional symptom scores in Black children and young people in 2020. Similarly, one general population cohort study in the UK found minoritised ethnic groups had better mental health outcomes during the initial phase of the pandemic compared to White youth (Miall et al., 2023). Another general population survey found that although all ethnic groups saw an increase in mental health problems between 2017 and July 2020, White children had a larger increase compared to minoritised ethnic groups (Vizard et al., 2020). Most clinical studies have found no association between ethnicity and mental health outcomes during the pandemic (Korczak et al., 2024; Pampati et al., 2023; Toseeb & Asbury, 2023), except Dvorsky et al. (2022) who found increased emotional symptoms in Black adolescents with ADHD compared to White and Asian peers between early to late 2020. Further research needs to use large representative clinical samples to understand the mechanisms that may underlie potential ethnic differences in mental health problems during the pandemic and how these differences may change temporally across pandemic‐related events.

Finally, we found that predictors for emotional and behavioural symptom trajectories varied across clinical groups. Specifically, ID diagnosis and age's effect on emotional and behavioural symptoms were only maintained in the ASD group. There may be diagnosis‐specific explanatory factors in the pandemic's impact on mental health in clinical groups, which was an emerging finding in a previous systematic review and meta‐analysis (Ching et al., 2025). However, the review had limited small‐sampled studies that investigated the effect of diagnosis. In our study, the reduced sample sizes in subgroup analysis may have also minimised true effects, especially for ID across main and subgroup analyses. Thus, our findings around ID should be interpreted cautiously and future research using larger samples should be conducted to investigate predictors of trajectories across heterogenous clinical groups, including those with ID.

Within the context of services, our findings do not clearly identify the impact of mental health service provisions on mental health during the pandemic. Our cohort specifically describes service users who were in services in the UK between 2019 and 2021, which may be capturing those that may have remained in services due to severity/risk or requiring monitoring for treatment. It is difficult to disentangle the effects of the pandemic and restrictions with naturalistic symptom trajectories during attendance to child and adolescent mental health services (CAMHS). In the UK, pandemic restrictions consisted of three national lockdowns between 2020 and 2021, repeated disruptions to school (closures, reopenings, and changes in the delivery of teaching), and increased difficulty in access to health and social care services (see Appendix S7 for more details on timeline of key pandemic events in the UK). Future work should compare trajectories between large clinical cohorts during the pandemic and account for service and treatment‐related factors to better capture causal effects of the pandemic.

Our study has several strengths by using a novel method linking survey and EHR data to examine longitudinal trajectories of a relatively large clinical group of children and young people attending mental health services in South London, supplementing data completeness by *z*‐transforming and combining outcomes and using rich sociodemographic and clinical data available. Adhering to recommendations for pandemic research in children and young people (Ching et al., 2024, 2025), we report means of outcomes across key characteristics, explore trajectories across diagnostic groups, use robust methods to address missing data, such as inverse probability weights, and attempt to explain heterogeneity in outcomes. Findings with and without weightings were largely consistent, suggesting results were robust to adjustment for potential selection bias in our analytic sample compared to the larger CAMHS cohort.

However, limitations should be considered. Firstly, attrition prevented examination of trajectories beyond 2021 and limited the analytic sample significantly compared to the larger CAMHS sample. Consequently, statistical analyses may be using smaller samples with wider CIs and reduced statistical power to detect true effects with high precision. Secondly, by the nature of the sampling frame, we were unable to capture children and young people with pre‐existing conditions who were not in services which may have biased our findings. Up to 70%–80% of young people with pre‐existing clinical need may not have accessed mental health services before and during the pandemic (Shidhaye, 2023) and delayed or absent treatment may contribute to poorer mental health outcomes (YoungMinds, 2018). Thus, our findings could potentially be underreporting negative changes over time. Thirdly, our study timepoints are large and encompassed multiple pandemic events. Although we addressed this by selecting outcome data close to each other to reduce between‐subject variation, variations were still present, and it was not possible to examine more granular changes resulting from specific pandemic events. Fourthly, due to insufficient data, we were unable to compare caregiver and self‐reported data. Additionally, due to inconsistence of measurement of outcomes across timepoints and having to use standardised emotional and behavioural symptoms scores comprised of two separate measures, we were only able to look at relative change across time. Potential stochastic variation arising from standardisation of two separate measures may have attenuated findings and underestimated effects. Thus, absolute changes across time should be investigated in future research to examine how mental health changed during the pandemic compared to prior.

These findings emphasise the stable yet high clinical needs of diverse groups of children and young people with pre‐existing mental health and neurodevelopmental conditions. Services providing mental healthcare to children and young people have had to continue support provisions during the pandemic and beyond to address stable yet elevated emotional and behavioural symptoms. Services may want to target vulnerable subgroups, including younger, female, and neurodivergent children and young people and those from deprived neighbourhoods. Clinicians may want to explore the longitudinal mental health impact of the pandemic with young people and their families to understand the mechanisms of relevant explanatory factors and inform intervention. These implications are relevant to address the long‐term impacts of this pandemic and useful for future crises.

Future research is needed to investigate longer‐term outcomes to understand emotional and behavioural symptom trajectories because of pandemic‐related restrictions and other mental health outcomes (e.g., self‐harm and suicide). Exploration of other potentially important factors are needed, such as school experiences, financial insecurity, and caregiver mental health (Ching et al., 2024; Parlatini et al., 2024). Qualitative studies should also be used to better understand mechanisms of change in predictors of mental health trajectories.

## CONCLUSIONS

Variations in trajectories of emotional and behavioural symptoms may be explained by sociodemographic and clinical characteristics. Future research should examine longer‐term impacts of the pandemic on emotional and behavioural symptoms and other mental health outcomes across diverse clinical groups, and the mechanisms of change in potential predictors.

## AUTHOR CONTRIBUTIONS

**Brian C. F. Ching**: Conceptualization (equal); data curation (lead); formal analysis (lead); investigation (lead); methodology (equal); project administration (lead); writing—original draft preparation (lead); writing—review and editing (lead). **Alice Wickersham**: Formal analysis (supporting); writing—review and editing (supporting). **Dominic Stringer**: Formal analysis (supporting); writing—review and editing (supporting). **Sarjhana Ragunathan Brindha**: Data curation (supporting); investigation (supporting); project administration (supporting); writing—review and editing (supporting). **Craig Colling**: Data curation (supporting); investigating (supporting); project administration (supporting); writing—review and editing (supporting). **Ruby Morton**: Formal analysis (supporting); writing—review and editing (supporting). **Valeria Parlatini**: Conceptualization (supporting); supervision (supporting); writing—review and editing (supporting). **Johnny Downs**: Conceptualization (equal); methodology (equal); supervision (equal); writing—original draft preparation (supporting); writing—review and editing (supporting). **Emily Simonoff**: Conceptualization (equal); methodology (equal); supervision (equal); writing—original draft preparation (supporting); writing—review and editing (supporting).

## CONFLICT OF INTEREST STATEMENT

The authors declare no conflicts of interest.

## ETHICAL CONSIDERATIONS

CRIS has research ethics approval for secondary analyses from South Central – Oxford C Research Ethics Committee (reference 23/SC/0257), and this specific project was approved by the SLaM CRIS oversight committee (number 23‐021) in March 2023. Caregivers and children and young people (with parental consent if below 16) were invited to complete the Maudsley Child and Young People Health and Experience Research (CYPHER) survey. The Maudsley CYPHER survey was developed and sent out as part of patient monitoring activities under the UK legal framework of Regulation 3(2)/3(3) of the Health Service Control of Patient Information 2002 (COPI). Databases used in this study operated on an opt‐out basis.

## Supporting information

Supporting Information S1

## Data Availability

The data that support the findings of this study are not publicly available but can be accessed with permissions from the South London and Maudsley NHS Foundation Trust.
