# Supplementary material for: Variations in trajectories of emotional and behavioural symptoms in children and young people with pre‐existing mental health and neurodevelopmental conditions before and during the COVID‐19 pandemic: A nested data linkage clinical cohort study
Source: JCPP Adv. 2026 Mar 17:e70115. Online ahead of print. doi: 10.1002/jcv2.70115 (PMC13339667; doi:10.1002/jcv2.70115)
Supplement: Supplementary file 1 — Supporting Information S1 [file JCV2-9999-e70115-s001.docx]

#### **Variations in trajectories of emotional and behavioural symptoms in children and young people with pre-existing mental health and neurodevelopmental conditions before and during the COVID-19 pandemic: a nested data linkage clinical cohort study**

**Supporting Information**

**Appendix S1.** The RECORD statement – checklist of items, extended from the STROBE statement, that should be reported in observational studies using routinely collected health data.

|  | **Item No.** | **STROBE items** | **Location in manuscript where items are reported** | **RECORD items** | **Location in manuscript where items are reported** |
| --- | --- | --- | --- | --- | --- |
| **Title and abstract** | | | | | |
|  | 1 | (a) Indicate the study’s design with a commonly used term in the title or the abstract (b) Provide in the abstract an informative and balanced summary of what was done and what was found | Title, P1. | RECORD 1.1: The type of data used should be specified in the title or abstract. When possible, the name of the databases used should be included.  RECORD 1.2: If applicable, the geographic region and timeframe within which the study took place should be reported in the title or abstract.  RECORD 1.3: If linkage between databases was conducted for the study, this should be clearly stated in the title or abstract. | P1.  Title, P1.  Title, P1. |
| **Introduction** | | | | | |
| Background rationale | 2 | Explain the scientific background and rationale for the investigation being reported | P2. |  |  |
| Objectives | 3 | State specific objectives, including any prespecified hypotheses | P1-2. |  |  |
| **Methods** | | | | | |
| Study Design | 4 | Present key elements of study design early in the paper | P3. |  |  |
| Setting | 5 | Describe the setting, locations, and relevant dates, including periods of recruitment, exposure, follow-up, and data collection | P3. |  |  |
| Participants | 6 | *(a) Cohort study* - Give the eligibility criteria, and the sources and methods of selection of participants. Describe methods of follow-up  *Case-control study* - Give the eligibility criteria, and the sources and methods of case ascertainment and control selection. Give the rationale for the choice of cases and controls  *Cross-sectional study* - Give the eligibility criteria, and the sources and methods of selection of participants  *(b) Cohort study* - For matched studies, give matching criteria and number of exposed and unexposed  *Case-control study* - For matched studies, give matching criteria and the number of controls per case | P3. | RECORD 6.1: The methods of study population selection (such as codes or algorithms used to identify subjects) should be listed in detail. If this is not possible, an explanation should be provided.  RECORD 6.2: Any validation studies of the codes or algorithms used to select the population should be referenced. If validation was conducted for this study and not published elsewhere, detailed methods and results should be provided.  RECORD 6.3: If the study involved linkage of databases, consider use of a flow diagram or other graphical display to demonstrate the data linkage process, including the number of individuals with linked data at each stage. | P3.  N/A.  P3, Figure 1. |
| Variables | 7 | Clearly define all outcomes, exposures, predictors, potential confounders, and effect modifiers. Give diagnostic criteria, if applicable. | P3-4. | RECORD 7.1: A complete list of codes and algorithms used to classify exposures, outcomes, confounders, and effect modifiers should be provided. If these cannot be reported, an explanation should be provided. | P3-4, Supplement. |
| Data sources/ measurement | 8 | For each variable of interest, give sources of data and details of methods of assessment (measurement).  Describe comparability of assessment methods if there is more than one group | P3-4. |  |  |
| Bias | 9 | Describe any efforts to address potential sources of bias | P3-4. |  |  |
| Study size | 10 | Explain how the study size was arrived at | P3. |  |  |
| Quantitative variables | 11 | Explain how quantitative variables were handled in the analyses. If applicable, describe which groupings were chosen, and why | P4-5. |  |  |
| Statistical methods | 12 | (a) Describe all statistical methods, including those used to control for confounding  (b) Describe any methods used to examine subgroups and interactions  (c) Explain how missing data were addressed  (d) *Cohort study* - If applicable, explain how loss to follow-up was addressed  *Case-control study* - If applicable, explain how matching of cases and controls was addressed  *Cross-sectional study* - If applicable, describe analytical methods taking account of sampling strategy  (e) Describe any sensitivity analyses | P4-5. |  |  |
| Data access and cleaning methods |  | .. |  | RECORD 12.1: Authors should describe the extent to which the investigators had access to the database population used to create the study population.  RECORD 12.2: Authors should provide information on the data cleaning methods used in the study. | P3.  P3-5. |
| Linkage |  | .. |  | RECORD 12.3: State whether the study included person-level, institutional-level, or other data linkage across two or more databases. The methods of linkage and methods of linkage quality evaluation should be provided. | P3. |
| **Results** | | | | | |
| Participants | 13 | (a) Report the numbers of individuals at each stage of the study (*e.g.*, numbers potentially eligible, examined for eligibility, confirmed eligible, included in the study, completing follow-up, and analysed)  (b) Give reasons for non-participation at each stage.  (c) Consider use of a flow diagram | P5, Figure 1. | RECORD 13.1: Describe in detail the selection of the persons included in the study (*i.e.,* study population selection) including filtering based on data quality, data availability and linkage. The selection of included persons can be described in the text and/or by means of the study flow diagram. | P5, Figure 1. |
| Descriptive data | 14 | (a) Give characteristics of study participants (*e.g.*, demographic, clinical, social) and information on exposures and potential confounders  (b) Indicate the number of participants with missing data for each variable of interest  (c) *Cohort study* - summarise follow-up time (*e.g.*, average and total amount) | P5, Supplement. |  |  |
| Outcome data | 15 | *Cohort study* - Report numbers of outcome events or summary measures over time  *Case-control study* - Report numbers in each exposure category, or summary measures of exposure  *Cross-sectional study* - Report numbers of outcome events or summary measures | P5-6. |  |  |
| Main results | 16 | (a) Give unadjusted estimates and, if applicable, confounder-adjusted estimates and their precision (e.g., 95% confidence interval). Make clear which confounders were adjusted for and why they were included  (b) Report category boundaries when continuous variables were categorized  (c) If relevant, consider translating estimates of relative risk into absolute risk for a meaningful time period | P5-P6. |  |  |
| Other analyses | 17 | Report other analyses done—e.g., analyses of subgroups and interactions, and sensitivity analyses | P6. |  |  |
| **Discussion** | | | | | |
| Key results | 18 | Summarise key results with reference to study objectives | P6. |  |  |
| Limitations | 19 | Discuss limitations of the study, taking into account sources of potential bias or imprecision. Discuss both direction and magnitude of any potential bias | P8. | RECORD 19.1: Discuss the implications of using data that were not created or collected to answer the specific research question(s). Include discussion of misclassification bias, unmeasured confounding, missing data, and changing eligibility over time, as they pertain to the study being reported. | P8. |
| Interpretation | 20 | Give a cautious overall interpretation of results considering objectives, limitations, multiplicity of analyses, results from similar studies, and other relevant evidence | P7-9. |  |  |
| Generalisability | 21 | Discuss the generalisability (external validity) of the study results | P7-9. |  |  |
| **Other Information** | | | | | |
| Funding | 22 | Give the source of funding and the role of the funders for the present study and, if applicable, for the original study on which the present article is based | Acknowledgements. |  |  |
| Accessibility of protocol, raw data, and programming code |  | .. |  | RECORD 22.1: Authors should provide information on how to access any supplemental information such as the study protocol, raw data, or programming code. | Acknowledgements. |

*Reference: Benchimol EI, Smeeth L, Guttmann A, Harron K, Moher D, Petersen I, Sørensen HT, von Elm E, Langan SM, the RECORD Working Committee. The REporting of studies Conducted using Observational Routinely-collected health Data (RECORD) Statement. *PLoS Medicine* 2015; in press.

*Checklist is protected under Creative Commons Attribution ([CC BY](http://creativecommons.org/licenses/by/4.0/)) license.

**Appendix S2.** The Maudsley CYPHER survey emotional and behavioural composite scores.

Separate emotional and behavioural composite scores were created and validated based on results from published factor analysis (Parlatini et al., 2023). The emotional score comprised of singular items assessing anhedonia, sadness, general worries, anxiety, fatigue, and loneliness. The behavioural score comprised of singular items assessing restlessness, inattention, irritability, and aggression. For each item, caregivers were asked about the severity of their child’s symptoms over the past two-weeks (e.g. for the general worries item, caregivers were asked “how worried was your child generally?”). Each item was scored between 1 to 5, with higher scores indicating more severe symptoms. Likert scores of individual items/symptoms were summed to generate a caregiver-reported total emotional (range 6-30) and behavioural score (range 4-20). See below for the individual items:

- How worried was your child generally? Not worried at all; slightly worried; moderately worried; very worried; extremely worried.
- How happy versus sad was your child? Very sad/depressed/unhappy; moderately sad/depressed/unhappy; neutral; moderately happy/cheerful; very happy/cheerful.
- How much has your child been able to enjoy his/her usual activities? Not at all; slightly; moderately; very much; a lot.
- How relaxed versus anxious was your child? Very relaxed/calm; moderately relaxed/calm; neutral; moderately nervous/anxious; very nervous/anxious.
- How fatigued or tired was your child? Not fatigued or tired at all; slightly fatigued or tired; moderately fatigued or tired; very fatigued or tired; extremely fatigued or tired.
- How lonely has your child been? Not lonely at all; slightly lonely; moderately lonely; very lonely; extremely lonely.
- How fidgety or restless was your child? Not restless at all; slightly restless; moderately restless; very restless; extremely restless.
- For their age, how well has your child been able to concentrate or focus? Very focused/attentive; moderately focused/attentive; neutral; moderately unfocused/distracted; very unfocused/distracted.
- How irritable or easily angered has your child been? Not irritable or easily angered at all; slightly irritable or easily angered; moderately irritable or easily angered; very irritable or easily angered; extremely irritable or easily angered.
- How physically aggressive towards others has your child been? No aggression at all; a little aggression, once or twice and not severe; some aggression but not severe; some aggression, including hurting others; frequent aggression, including hurting others.

**References:**

Parlatini V, Frangou L, Zhang S, Epstein S, Morris A, Grant C, Zalewski L, Jewell A, Velupillai S, Simonoff E, Downs J. Emotional and behavioral outcomes among youths with mental disorders during the first Covid lockdown and school closures in England: a large clinical population study using health care record integrated surveys. Social Psychiatry and Psychiatric Epidemiology. 2024 Jan;59(1):175-86.

**Appendix S3.** Emotional and behavioural symptom score standardisation and construction.

| Timepoint | Measure(s) | Standardisation* | Composite |
| --- | --- | --- | --- |
| Pre-pandemic (2019) | SDQ | SDQ raw scores z-standardised within pre-pandemic separately for emotional and behavioural symptoms. | N/A (SDQ z-scores directly used). |
| 2020 | SDQ  Maudsley CYPHER survey | SDQ raw scores z-standardised within 2020 separately for emotional and behavioural symptoms.  Maudsley CYPHER raw scores z-standardised within 2020 separately for emotional and behavioural symptoms. | Average of SDQ and Maudsley CYPHER z-scores for emotional and behavioural symptoms separately. |
| 2021 | SDQ  Maudsley CYPHER survey | SDQ raw scores z-standardised within 2021 separately for emotional and behavioural symptoms.  Maudsley CYPHER raw scores z-standardised within 2021 separately for emotional and behavioural symptoms. | Average of SDQ and Maudsley CYPHER z-scores for emotional and behavioural symptoms separately. |

*Mean = 0 and standard deviation (SD) = 1.

**Appendix S4.** Change in z-score reflected in change in units in SDQ and Maudsley CYPHER emotional and behavioural symptom scores.

As only SDQs were available at pre-pandemic, we were unable to standardise z-scores using SDQs in 2019/baseline as the reference. However, z-scores are comparable; A change in 0.1 in emotional symptoms z-score is the same as 0.41 in SDQ in pre-pandemic, 0.49 in Maudsley CYPHER in 2020, 0.44 in SDQ in 2020, 0.47 in Maudsley CYPHER in 2021, and 0.45 in SDQ in 2021. A change in 0.1 in behavioural symptoms z-score is the same as 0.44 in SDQ in pre-pandemic, 0.38 in Maudsley CYPHER in 2020, 0.43 in SDQ in 2020, 0.34 in Maudsley CYPHER in 2021, and 0.39 in SDQ in 2021. Mean scores of combined outcomes are also not significantly different from each other across timepoints (see Table S2).

We do not run into the problems that may arise from z-standardisation (Moeller, 2015):

1. Standardising repeated measures within individuals impedes examining mean-level differences between individuals – we did not do this.
2. Standardisation across individuals within measurement time points impedes examining mean level changes from one time point – we focused on relative change longitudinally across characteristics. We have demonstrated that z-score changes are comparable to consistent raw SDQ and Maudsley CYPHER emotional and behavioural score change over time. We also looked at differences in outcomes across time by predictors and did not standardise outcomes within groups (e.g. within ASD, ADHD, or emotional disorders groups); all groups would need to deteriorate consistently in the same direction to mitigate across group differences over time.
3. Standardisation across individuals across time points – we did not do this.
4. Standardisation across individuals within age groups/cohorts impedes studying age differences at given time points – we did not do this.
5. Plotting group differences using z-scores often makes eventually small differences look big, compared to a graph displaying the complete original response scale and raw scores – we did not do this.

**References:**

Moeller J. A word on standardization in longitudinal studies: don't. Frontiers in psychology. 2015 Sep 15;6:1389.

**Appendix S5.** Sample characteristics of children and young people in the analytic sample.

The most represented diagnoses in our CAMHS population were: Attention Deficit Hyperactivity Disorder-ADHD (i.e. hyperkinetic disorders, ICD-10 codes F90.0, F90.1, F90.2, F90.8, F90.9); Autism Spectrum Disorder-ASD (F84.0, F84.1, F84.5, F84.9); and emotional disorders, which include depressive disorders, anxiety disorders, Post-Traumatic Stress Disorder-PTSD, and Obsessive-Compulsive Disorder-OCD (F32, F33, F34, F38, F40-49, F93, F94). For group comparisons, as co-occurring disorders are frequent, diagnoses were organised according to a hierarchy according to the main treatment pathway: children and young people were assigned to the ASD group regardless of whether they also had ADHD and/or emotional disorders, whilst CYP were assigned to the ADHD group regardless of whether they had secondary emotional disorders.

**Appendix S6.** Linear mixed model random effects structure.

We compared a model including random intercepts for individuals to a model that additionally included random slopes for year using a likelihood ratio test. Random slopes did not significantly improve model fit (p > .05) and were therefore not included. Models with unstructured covariance matrices for random effects did not converge and were also not included. All reported models include only random intercepts for individuals.

**Appendix S7.** Brief timeline of key COVID-19 events in the UK.

March 2020 – first national lockdown, school closure, strict restrictions.

June 2020 – lockdown lifts, phased re-opening of schools in England, relaxing of restrictions and 2m social distancing rule.

July-August 2020 – local lockdowns implemented, lockdown restrictions ease, reopening of restaurants, hairdressers, pubs, indoor theatres, bowling alleys, and soft play.

September 2020 – rule of six social gatherings, new restrictions, 10pm curfew for restaurants and bars.

November 2020 – second national lockdown, three household meet for Christmas.

December 2020 – lockdown lifts, tiered restrictions, vaccines for adults available.

January 2021 – third national lockdown.

March 2021 – schools reopen, rule of six social gatherings back.

July 2021 – most restrictions relaxed.

December 2021 – NHS Covid pass and face masks mandatory, advises vaccine for 5-11 year old children who are clinically vulnerable.

January 2022 – Omicron variant.

February 2022 – all restrictions lifted.

April 2022 – vaccines for children available.

See <https://gds.blog.gov.uk/2022/07/25/2-years-of-covid-19-on-gov-uk/> for more details.

**Table S1.** Raw emotional and behavioural symptoms scores before z-transformation across time.

| **Symptom** | **Pre-pandemic**  Mean (SD) | **2020**  Mean (SD) | **2021**  Mean (SD) |
| --- | --- | --- | --- |
| Emotional symptoms | SDQ (n=388): 13.19 (4.14) | SDQ (n=86): 12.76 (4.37) | SDQ (n=100): 13.35 (4.45) |
|  |  | Pre-pandemic vs 2020: t(85) = 0.69, p = .491 | Pre-pandemic vs 2021: t(99) = 0.14, p = .891  2020 vs 2021:  t(21) = -2.48, p = .022 |
|  |  | Maudsley CYPHER (n=268): 18.08 (4.92) | Maudsley CYPHER (n=118): 17.55 (4.73) |
|  |  |  | 2020 vs 2021:  t(82) = 1.20, p = .233 |
| Behavioural symptoms | SDQ (n=388): 12.66 (4.39) | SDQ (n=86): 12.72 (4.35) | SDQ (n=100): 12.69 (3.90) |
|  |  | Pre-pandemic vs 2020: t(85) = -0.61, p = .541 | Pre-pandemic vs 2021: t(99) = 1.77, p = .080  2020 vs 2021:  t(21) = -0.63, p = .536 |
|  |  | Maudsley CYPHER (n=265): 13.11 (3.78) | Maudsley CYPHER (n=118): 12.96 (3.36) |
|  |  |  | 2020 vs 2021:  t(82) = -0.72, p = .471 |

Maudsley CYPHER: Maudsley Child and Young People Health and Experience Research (CYPHER) survey; SD: standard deviation; SDQ: The Strengths and Difficulties Questionnaire.

Note: This table represents descriptive data of the analytic sample, which included young people within the larger CAMHS (child and adolescent mental health services) cohort with complete sociodemographic and clinical characteristic data and any emotional and behavioural symptoms data in pre-pandemic and 2020 and/or 2021; Paired t-tests were conducted to produce change scores indicating mean differences of emotional and behavioural symptoms scores across timepoints.

**Table S2.** Raw emotional and behavioural symptoms scores before z-transformation by age.

| **Symptom** | **Age group** | **Pre-pandemic**  Mean (SD) | **2020**  Mean (SD) | **2021**  Mean (SD) |
| --- | --- | --- | --- | --- |
| Emotional symptoms | Primary school | SDQ (n=191): 13.15 (4.12) | SDQ (n=39): 12.05 (3.87) | SDQ (n=58): 13.64 (3.92) |
|  |  |  | Maudsley CYPHER (n=139): 17.89 (4.91) | Maudsley CYPHER (n=54): 17.44 (5.00) |
|  | Secondary school | SDQ (n=126): 13.02 (4.14) | SDQ (n=26): 13.04 (4.23) | SDQ (n=28): 12.89 (4.98) |
|  |  |  | Maudsley CYPHER (n=84): 18.18 (4.78) | Maudsley CYPHER (n=43): 17.47 (4.52) |
|  | College | SDQ (n=71): 13.59 (4.23) | SDQ (n=21): 13.71 (5.32) | SDQ (n=14): 13.07 (5.58) |
|  |  |  | Maudsley CYPHER (n=45): 18.47 (5.27) | Maudsley CYPHER (n=21): 18.00 (4.65) |
| Behavioural symptoms | Primary school | SDQ (n=191): 12.99 (4.38) | SDQ (n=39): 13.33 (4.10) | SDQ (n=58): 13.09 (3.61) |
|  |  |  | Maudsley CYPHER (n=138): 14.17 (3.61) | Maudsley CYPHER (n=54): 13.91 (3.25) |
|  | Secondary school | SDQ (n=126): 12.80 (4.14) | SDQ (n=26): 12.50 (4.53) | SDQ (n=28): 12.04 (4.32) |
|  |  |  | Maudsley CYPHER (n=83): 12.10 (3.84) | Maudsley CYPHER (n=43): 12.79 (3.00) |
|  | College | SDQ (n=71): 11.52 (4.69) | SDQ (n=21): 11.86 (4.61) | SDQ (n=14): 12.36 (4.29) |
|  |  |  | Maudsley CYPHER (n=44): 11.70 (3.24) | Maudsley CYPHER (n=21): 10.86 (3.48) |

Maudsley CYPHER: Maudsley Child and Young People Health and Experience Research (CYPHER) survey; SD: standard deviation; SDQ: The Strengths and Difficulties Questionnaire.

Note: This table represents descriptive data of the analytic sample, which included young people within the larger CAMHS (child and adolescent mental health services) cohort with complete sociodemographic and clinical characteristic data and any emotional and behavioural symptoms data in pre-pandemic and 2020 and/or 2021; Chi-squared tests were conducted to produce change scores indicating mean differences of emotional and behavioural symptoms scores across groups.

**Table S3.** Raw emotional and behavioural symptoms scores before z-transformation by sex.

| **Symptom** | **Sex** | **Pre-pandemic**  Mean (SD) | **2020**  Mean (SD) | **2021**  Mean (SD) |
| --- | --- | --- | --- | --- |
| Emotional symptoms | Male | SDQ (n=242): 12.90 (4.18) | SDQ (n=49): 12.39 (4.62) | SDQ (n=66): 13.21 (4.87) |
|  |  |  | Maudsley CYPHER (n=166): 17.77 (4.76) | Maudsley CYPHER (n=76): 17.18 (4.79) |
|  | Female | SDQ (n=146): 13.67 (4.05) | SDQ (n=37): 13.24 (4.02) | SDQ (n=34): 13.62 (3.57) |
|  |  |  | Maudsley CYPHER (n=102): 18.58 (5.16) | Maudsley CYPHER (n=42): 18.21 (4.59) |
| Behavioural symptoms | Male | SDQ (n=242): 13.05 (4.16) | SDQ (n=49): 13.06 (4.15) | SDQ (n=66): 12.67 (4.28) |
|  |  |  | Maudsley CYPHER (n=164): 13.27 (3.83) | Maudsley CYPHER (n=76): 13.22 (3.38) |
|  | Female | SDQ (n=146): 12.01 (4.69) | SDQ (n=37): 12.27 (4.62) | SDQ (n=34): 12.74 (3.11) |
|  |  |  | Maudsley CYPHER (n=101): 12.85 (3.70) | Maudsley CYPHER (n=42): 12.48 (3.33) |

Maudsley CYPHER: Maudsley Child and Young People Health and Experience Research (CYPHER) survey; SD: standard deviation; SDQ: The Strengths and Difficulties Questionnaire.

Note: This table represents descriptive data of the analytic sample, which included young people within the larger CAMHS (child and adolescent mental health services) cohort with complete sociodemographic and clinical characteristic data and any emotional and behavioural symptoms data in pre-pandemic and 2020 and/or 2021; Chi-squared tests were conducted to produce change scores indicating mean differences of emotional and behavioural symptoms scores across groups.

**Table S4.** Raw emotional and behavioural symptoms scores before z-transformation by ethnicity.

| **Symptom** | **Ethnicity** | **Pre-pandemic**  Mean (SD) | **2020**  Mean (SD) | **2021**  Mean (SD) |
| --- | --- | --- | --- | --- |
| Emotional symptoms | White | SDQ (n=198): 13.29 (3.93) | SDQ (n=48): 13.85 (4.37) | SDQ (n=26): 13.31 (4.04) |
|  |  |  | Maudsley CYPHER (n=133): 18.85 (5.01) | Maudsley CYPHER (n=66): 18.09 (4.44) |
|  | Black | SDQ (n=59): 13.39 (3.95) | SDQ (n=16): 11.56 (3.97) | SDQ (n=53): 13.42 (4.72) |
|  |  |  | Maudsley CYPHER (n=38): 16.50 (4.65) | Maudsley CYPHER (n=11): 16.09 (4.39) |
|  | Asian, Mixed, and Other | SDQ (n=62): 13.03 (4.67) | SDQ (n=12): 11.75 (3.93) | SDQ (n=21): 13.24 (4.45) |
|  |  |  | Maudsley CYPHER (n=43): 18.28 (4.59) | Maudsley CYPHER (n=16): 19.25 (4.16) |
|  | Not stated | SDQ (n=69): 12.87 (4.46) | SDQ (n=10): 10.60 (4.48) | SDQ (n=21): 13.24 (4.45) |
|  |  |  | Maudsley CYPHER (n=54): 17.13 (4.82) | Maudsley CYPHER (n=25): 15.68 (5.44) |
| Behavioural symptoms | White | SDQ (n=198): 12.59 (4.42) | SDQ (n=48): 12.56 (4.12) | SDQ (n=46): 12.48 (4.31) |
|  |  |  | Maudsley CYPHER (n=131): 13.64 (3.59) | Maudsley CYPHER (n=66): 13.29 (3.29) |
|  | Black | SDQ (n=59): 12.73 (4.50) | SDQ (n=16): 12.63 (4.79) | SDQ (n=16): 13.50 (4.27) |
|  |  |  | Maudsley CYPHER (n=38): 12.26 (3.18) | Maudsley CYPHER (n=11): 12.45 (2.54) |
|  | Asian, Mixed, and Other | SDQ (n=62): 12.77 (4.33) | SDQ (n=12): 14.17 (4.28) | SDQ (n=21): 12.57 (3.08) |
|  |  |  | Maudsley CYPHER (n=43): 12.51 (4.27) | Maudsley CYPHER (n=16): 13.31 (3.32) |
|  | Not stated | SDQ (n=69): 12.70 (4.33) | SDQ (n=10): 11.90 (5.07) | SDQ (n=17): 12.65 (3.52) |
|  |  |  | Maudsley CYPHER (n=53): 12.91 (4.12) | Maudsley CYPHER (n=25): 12.08 (3.87) |

Maudsley CYPHER: Maudsley Child and Young People Health and Experience Research (CYPHER) survey; SD: standard deviation; SDQ: The Strengths and Difficulties Questionnaire.

Note: This table represents descriptive data of the analytic sample, which included young people within the larger CAMHS (child and adolescent mental health services) cohort with complete sociodemographic and clinical characteristic data and any emotional and behavioural symptoms data in pre-pandemic and 2020 and/or 2021; Chi-squared tests were conducted to produce change scores indicating mean differences of emotional and behavioural symptoms scores across groups.

**Table S5.** Raw emotional and behavioural symptoms scores before z-transformation by neighbourhood deprivation.

| **Symptom** | **Deprivation** | **Pre-pandemic**  Mean (SD) | **2020**  Mean (SD) | **2021**  Mean (SD) |
| --- | --- | --- | --- | --- |
| Emotional symptoms | Not deprived | SDQ (n=312): 12.97 (4.30) | SDQ (n=65): 12.66 (4.74) | SDQ (n=74): 13.08 (4.55) |
|  |  |  | Maudsley CYPHER (n=216): 17.97 (4.93) | Maudsley CYPHER (n=98): 17.26 (4.79) |
|  | Deprived | SDQ (n=76): 14.08 (3.27) | SDQ (n=21): 13.05 (2.99) | SDQ (n=26): 14.12 (4.14) |
|  |  |  | Maudsley CYPHER (n=52): 18.52 (4.91) | Maudsley CYPHER (n=20): 19.00 (4.22) |
| Behavioural symptoms | Not deprived | SDQ (n=312): 12.40 (4.39) | SDQ (n=65): 12.63 (4.09) | SDQ (n=74): 12.38 (3.88) |
|  |  |  | Maudsley CYPHER (n=214): 13.08 (3.76) | Maudsley CYPHER (n=98): 12.76 (3.41) |
|  | Deprived | SDQ (n=76): 13.72 (4.25) | SDQ (n=21): 13.00 (5.17) | SDQ (n=26): 13.58 (3.90) |
|  |  |  | Maudsley CYPHER (n=51): 13.24 (3.89) | Maudsley CYPHER (n=20): 13.95 (3.03) |

Maudsley CYPHER: Maudsley Child and Young People Health and Experience Research (CYPHER) survey; SD: standard deviation; SDQ: The Strengths and Difficulties Questionnaire.

Note: This table represents descriptive data of the analytic sample, which included young people within the larger CAMHS (child and adolescent mental health services) cohort with complete sociodemographic and clinical characteristic data and any emotional and behavioural symptoms data in pre-pandemic and 2020 and/or 2021; Chi-squared tests were conducted to produce change scores indicating mean differences of emotional and behavioural symptoms scores across groups.

**Table S6.** Raw emotional and behavioural symptoms scores before z-transformation by primary diagnosis.

| **Symptom** | **Diagnosis** | **Pre-pandemic**  Mean (SD) | **2020**  Mean (SD) | **2021**  Mean (SD) |
| --- | --- | --- | --- | --- |
| Emotional symptoms | ADHD | SDQ (n=84): 11.61 (4.23) | SDQ (n=22): 11.55 (4.38) | SDQ (n=26): 13.31 (4.04) |
|  |  |  | Maudsley CYPHER (n=54): 15.65 (3.68) | Maudsley CYPHER (n=28): 16.04 (5.17) |
|  | ASD | SDQ (n=197): 14.11 (3.95) | SDQ (n=31): 14.87 (3.88) | SDQ (n=53): 13.42 (4.72) |
|  |  |  | Maudsley CYPHER (n=148): 18.92 (5.10) | Maudsley CYPHER (n=61): 18.15 (4.53) |
|  | Emotional disorders | SDQ (n=107): 12.73 (4.00) | SDQ (n=33): 11.58 (4.15) | SDQ (n=21): 13.24 (4.45) |
|  |  |  | Maudsley CYPHER (n=66): 18.18 (4.79) | Maudsley CYPHER (n=29): 17.76 (4.54) |
| Behavioural symptoms | ADHD | SDQ (n=84): 13.40 (4.48) | SDQ (n=22): 14.32 (4.35) | SDQ (n=26): 13.88 (3.78) |
|  |  |  | Maudsley CYPHER (n=54): 13.28 (3.53) | Maudsley CYPHER (n=28): 13.71 (3.24) |
|  | ASD | SDQ (n=197): 13.14 (4.15) | SDQ (n=31): 12.94 (3.78) | SDQ (n=53): 12.26 (3.89) |
|  |  |  | Maudsley CYPHER (n=146): 13.90 (3.64) | Maudsley CYPHER (n=61): 13.34 (3.42) |
|  | Emotional disorders | SDQ (n=107): 11.19 (4.44) | SDQ (n=33): 11.45 (4.58) | SDQ (n=21): 12.29 (3.98) |
|  |  |  | Maudsley CYPHER (n=65): 11.22 (3.68) | Maudsley CYPHER (n=29): 11.41 (2.97) |

Maudsley CYPHER: Maudsley Child and Young People Health and Experience Research (CYPHER) survey; SD: standard deviation; SDQ: The Strengths and Difficulties Questionnaire.

Note: This table represents descriptive data of the analytic sample, which included young people within the larger CAMHS (child and adolescent mental health services) cohort with complete sociodemographic and clinical characteristic data and any emotional and behavioural symptoms data in pre-pandemic and 2020 and/or 2021; Chi-squared tests were conducted to produce change scores indicating mean differences of emotional and behavioural symptoms scores across groups.

**Table S7.** Raw emotional and behavioural symptoms scores before z-transformation by intellectual disability (ID).

| **Symptom** | **ID status** | **Pre-pandemic**  Mean (SD) | **2020**  Mean (SD) | **2021**  Mean (SD) |
| --- | --- | --- | --- | --- |
| Emotional symptoms | No ID | SDQ (n=379): 13.16 (4.17) | SDQ (n=85): 12.76 (4.39) | SDQ (n=99): 13.45 (4.35) |
|  |  |  | Maudsley CYPHER (n=261): 18.13 (4.92) | Maudsley CYPHER (n=116): 17.68 (4.65) |
|  | ID | Supressed | Supressed | Supressed |
| Behavioural symptoms | No ID | SDQ (n=379): 12.64 (4.39) | SDQ (n=85): 12.74 (4.37) | SDQ (n=99): 12.73 (3.91) |
|  |  |  | Maudsley CYPHER (n=258): 13.11 (3.82) | Maudsley CYPHER (n=116): 13.01 (3.36) |
|  | ID | Supressed | Supressed | Supressed |

Maudsley CYPHER: Maudsley Child and Young People Health and Experience Research (CYPHER) survey; SD: standard deviation; SDQ: The Strengths and Difficulties Questionnaire.

Note: This table represents descriptive data of the analytic sample, which included young people within the larger CAMHS (child and adolescent mental health services) cohort with complete sociodemographic and clinical characteristic data and any emotional and behavioural symptoms data in pre-pandemic and 2020 and/or 2021; Chi-squared tests were conducted to produce change scores indicating mean differences of emotional and behavioural symptoms scores across groups. Cells representing N>10 has not been reported due to potentially disclosive cell counts in line with statistical disclosure guidance.

**Table S8.** Sample characteristics of children and young people in the larger CAMHS cohort versus the analytic sample.

|  | **CAMHS cohort** (n=5,386) | **Analytic sample** (n=388) |
| --- | --- | --- |
| **Median (SD), range** |  |  |
| Age | 13.69 (3.25), 4.45 to 19.43 | 12.32 (3.23), 5.44 to 18.44 |
| **n (%)** |  |  |
| Sex |  |  |
| Male | 3,056 (56.74%) | 242 (62.37%) |
| Female | 2,308 (42.85%) | 146 (37.63%) |
| Not stated | 22 (0.41%) | 0 (0%) |
| Ethnicity |  |  |
| White | 2,482 (46.08%) | 198 (51.03%) |
| Black | 1,151 (21.37%) | 59 (15.21%) |
| Asian, Mixed, and Other | 805 (14.95%) | 62 (15.98%) |
| Not stated | 947 (17.59%) | 69 (17.78%) |
| Neighbourhood deprivation |  |  |
| Not deprived | 4,191 (77.81%) | 312 (80.41%) |
| Deprived | 1,049 (19.48%) | 76 (19.59%) |
| Not stated | 146 (2.71%) | 0 (0%) |
| Main three diagnosis |  |  |
| ADHD | 1,286 (23.88%) | 84 (21.65%) |
| ASD | 1,372 (25.47%) | 197 (50.77%) |
| Emotional disorders | 1,015 (18.85%) | 107 (27.58%) |
| Not stated | 1,713 (31.80%) | 0 (0%) |
| Intellectual disability |  |  |
| No/not stated | 5,175 (96.08%) | Suppressed |
| Yes | 211 (3.92%) | Suppressed |

ADHD: attention-deficit/hyperactivity disorder; ASD: autism spectrum disorder; CAMHS: child and adolescent mental health services; SD: standard deviation.

Note: The analytic sample included young people within the larger CAMHS cohort with complete sociodemographic and clinical characteristic data and any emotional and behavioural symptoms data in pre-pandemic and 2020 and/or 2021; The intellectual disability variable in the analytic sample has not been reported due to potentially disclosive cell counts in line with statistical disclosure guidance.

**Table S9.** Estimated trajectories of emotional symptoms with interactions for time with sex, ethnicity, neighbourhood deprivation, primary diagnosis, and ID diagnosis between pre-pandemic and 2021 with inverse probability weights.

|  | **Fixed effect** |
| --- | --- |
| **Predictor** | \| b \| SE \| Z \| 95% CI \| p \| \| --- \| --- \| --- \| --- \| --- \| |
| Year (slope)* |  |
| Pre-pandemic | Reference |
| 2020 | \| 0.32 \| 0.15 \| 2.15 \| 0.03 to 0.61 \| .032 \| \| --- \| --- \| --- \| --- \| --- \| |
| 2021 | \| 0.17 \| 0.21 \| 0.79 \| -0.25 to 0.58 \| .432 \| \| --- \| --- \| --- \| --- \| --- \| |
| Age at start of pandemic | \| 0.02 \| 0.01 \| 1.42 \| -0.01 to 0.05 \| .156 \| \| --- \| --- \| --- \| --- \| --- \| |
| Sex |  |
| Male | Reference |
| Female | \| 0.17 \| 0.13 \| 1.36 \| -0.08 to 0.42 \| .173 \| \| --- \| --- \| --- \| --- \| --- \| |
| Ethnicity |  |
| White | Reference |
| Black | \| 0.18 \| 0.15 \| 1.20 \| -0.11 to 0.46 \| .231 \| \| --- \| --- \| --- \| --- \| --- \| |
| Asian, Mixed, or Other | \| 0.00 \| 0.15 \| 0.01 \| -0.30 to 0.30 \| .989 \| \| --- \| --- \| --- \| --- \| --- \| |
| Not stated | \| -0.05 \| 0.15 \| -0.34 \| -0.34 to 0.24 \| .731 \| \| --- \| --- \| --- \| --- \| --- \| |
| Neighbourhood deprivation |  |
| Not deprived | Reference |
| Deprived | \| 0.30 \| 0.12 \| 2.42 \| 0.06 to 0.54 \| .016 \| \| --- \| --- \| --- \| --- \| --- \| |
| Primary diagnosis |  |
| Emotional disorders | Reference |
| ADHD | \| -0.15 \| 0.17 \| -0.86 \| -0.48 to 0.19 \| .387 \| \| --- \| --- \| --- \| --- \| --- \| |
| ASD | \| 0.44 \| 0.13 \| 3.31 \| 0.18 to 0.70 \| .001 \| \| --- \| --- \| --- \| --- \| --- \| |
| Intellectual disability |  |
| No or not stated | Reference |
| Yes | \| 0.16 \| 0.21 \| 0.77 \| -0.25 to 0.57 \| .444 \| \| --- \| --- \| --- \| --- \| --- \| |
| Year#Sex |  |
| 2020#Female | \| 0.01 \| 0.16 \| 0.07 \| -0.30 to 0.32 \| .948 \| \| --- \| --- \| --- \| --- \| --- \| |
| 2021#Female | \| 0.07 \| 0.22 \| 0.30 \| -0.36 to 0.49 \| .763 \| \| --- \| --- \| --- \| --- \| --- \| |
| Year#Ethnicity |  |
| 2020#Black | \| -0.45 \| 0.18 \| -2.45 \| -0.81 to -0.09 \| .014 \| \| --- \| --- \| --- \| --- \| --- \| |
| 2020#Asian, Mixed, and Other | \| -0.20 \| 0.19 \| -1.04 \| -0.58 to 0.18 \| .297 \| \| --- \| --- \| --- \| --- \| --- \| |
| 2020#Not stated | \| -0.46 \| 0.18 \| -2.61 \| -0.81 to -0.11 \| .009 \| \| --- \| --- \| --- \| --- \| --- \| |
| 2021#Black | \| -0.32 \| 0.27 \| -1.21 \| -0.84 to 0.20 \| .227 \| \| --- \| --- \| --- \| --- \| --- \| |
| 2021#Asian, Mixed, and Other | \| 0.11 \| 0.23 \| 0.48 \| -0.34 to 0.56 \| .628 \| \| --- \| --- \| --- \| --- \| --- \| |
| 2021#Not stated | \| -0.15 \| 0.20 \| -0.74 \| -0.53 to 0.24 \| .459 \| \| --- \| --- \| --- \| --- \| --- \| |
| Year#Neighbourhood deprivation |  |
| 2020#Deprived | \| -0.18 \| 0.15 \| -1.20 \| -0.47 to 0.11 \| .231 \| \| --- \| --- \| --- \| --- \| --- \| |
| 2021#Deprived | \| -0.24 \| 0.26 \| -0.93 \| -0.75 to 0.27 \| .352 \| \| --- \| --- \| --- \| --- \| --- \| |
| Year#Primary diagnosis |  |
| 2020#ADHD | \| -0.20 \| 0.20 \| -1.00 \| -0.59 to 0.19 \| .319 \| \| --- \| --- \| --- \| --- \| --- \| |
| 2020#ASD | \| -0.17 \| 0.16 \| -1.03 \| -0.48 to 0.15 \| .302 \| \| --- \| --- \| --- \| --- \| --- \| |
| 2021#ADHD | \| 0.17 \| 0.25 \| 0.68 \| -0.32 to 0.67 \| .494 \| \| --- \| --- \| --- \| --- \| --- \| |
| 2021#ASD | \| -0.21 \| 0.22 \| -0.95 \| -0.64 to 0.22 \| .341 \| \| --- \| --- \| --- \| --- \| --- \| |
| Year#Intellectual disability |  |
| 2020#Yes | \| -0.94 \| 0.35 \| -2.71 \| -1.62 to -0.26 \| .007 \| \| --- \| --- \| --- \| --- \| --- \| |
| 2021#Yes | \| -1.84 \| 0.26 \| -7.06 \| -2.34 to -1.33 \| .000 \| \| --- \| --- \| --- \| --- \| --- \| |
| Intercept (reference groups) | \| -0.52 \| 0.23 \| -2.23 \| -0.98 to -0.06 \| .026 \| \| --- \| --- \| --- \| --- \| --- \| |
|  |  |
|  | **Random effect** |
|  | \| Estimate \| SE \| 95% CI \| \| --- \| --- \| --- \| |
| Variance (intercept) | \| 0.53 \| 0.04 \| 0.45 to 0.61 \| \| --- \| --- \| --- \| |
| Variance (residual) | \| 0.32 \| 0.03 \| 0.27 to 0.37 \| \| --- \| --- \| --- \| |
| ICC | \| 0.63 \| 0.03 \| 0.57 to 0.68 \| \| --- \| --- \| --- \| |

ADHD: attention-deficit/hyperactivity disorder; ASD: autism spectrum disorder; ID: intellectual disability.

b: fixed effect coefficient; ICC: intraclass correlation coefficient; SE: standard error; z: z-score; 95% CI: 95% confidence interval; p: p-value; #: interaction term.

*Outcomes were z-standardised within each timepoint (pre-pandemic, 2020, 2021); therefore, coefficients for time reflect relative changes in symptoms.

Note: This table represents data from linear mixed modelling using the analytic sample, which included young people within the larger CAMHS (child and adolescent mental health services) cohort with complete sociodemographic and clinical characteristic data and any emotional and behavioural symptoms data in pre-pandemic and 2020 and/or 2021; Inverse probability weights were applied to the analysis, which were based on logistic regressions examining whether study predictors were associated with inclusion in the analytic sample (having emotional and behavioural symptoms data in pre-pandemic and 2020-2021). From these models, each participant’s predicted probability of inclusion was obtained, and inverse probability weights were derived as the reciprocal of these probabilities.

**Table S10.** Estimated trajectories of behavioural symptoms with interactions for time with age and primary diagnosis between pre-pandemic and 2021 with inverse probability weights.

|  | **Fixed effect** |
| --- | --- |
| **Predictor** | \| b \| SE \| Z \| 95% CI \| p \| \| --- \| --- \| --- \| --- \| --- \| |
| Year (slope)* |  |
| Pre-pandemic | Reference |
| 2020 | \| 0.23 \| 0.45 \| 0.51 \| -0.65 to 1.11 \| .612 \| \| --- \| --- \| --- \| --- \| --- \| |
| 2021 | \| 0.34 \| 0.48 \| 0.70 \| -0.61 to 1.28 \| .484 \| \| --- \| --- \| --- \| --- \| --- \| |
| Age at start of pandemic | \| -0.02 \| 0.02 \| -1.04 \| -0.06 to 0.02 \| .301 \| \| --- \| --- \| --- \| --- \| --- \| |
| Sex |  |
| Male | Reference |
| Female | \| -0.01 \| 0.08 \| -0.16 \| -0.18 to 0.15 \| .873 \| \| --- \| --- \| --- \| --- \| --- \| |
| Ethnicity |  |
| White | Reference |
| Black | \| -0.03 \| 0.10 \| -0.33 \| -0.24 to 0.17 \| .739 \| \| --- \| --- \| --- \| --- \| --- \| |
| Asian, Mixed, or Other | \| -0.00 \| 0.11 \| -0.01 \| -0.22 to 0.21 \| .994 \| \| --- \| --- \| --- \| --- \| --- \| |
| Not stated | \| -0.11 \| 0.11 \| -1.06 \| -0.32 to 0.09 \| .289 \| \| --- \| --- \| --- \| --- \| --- \| |
| Neighbourhood deprivation |  |
| Not deprived | Reference |
| Deprived | \| 0.13 \| 0.10 \| 1.34 \| -0.06 to 0.32 \| .180 \| \| --- \| --- \| --- \| --- \| --- \| |
| Primary diagnosis |  |
| Emotional disorders | Reference |
| ADHD | \| 0.44 \| 0.18 \| 2.43 \| 0.09 to 0.80 \| .015 \| \| --- \| --- \| --- \| --- \| --- \| |
| ASD | \| 0.42 \| 0.13 \| 3.22 \| 0.17 to 0.68 \| .001 \| \| --- \| --- \| --- \| --- \| --- \| |
| Intellectual disability |  |
| No or not stated | Reference |
| Yes | \| -0.00 \| 0.17 \| -0.02 \| -0.35 to 0.34 \| .981 \| \| --- \| --- \| --- \| --- \| --- \| |
| Year#Age at start of pandemic |  |
| 2020 | \| -0.02 \| 0.03 \| -0.68 \| -0.08 to 0.04 \| .498 \| \| --- \| --- \| --- \| --- \| --- \| |
| 2021 | \| -0.03 \| 0.03 \| -0.93 \| -0.09 to 0.03 \| .352 \| \| --- \| --- \| --- \| --- \| --- \| |
| Year#Primary diagnosis |  |
| 2020#ADHD | \| -0.09 \| 0.28 \| -0.31 \| -0.63 to 0.46 \| .754 \| \| --- \| --- \| --- \| --- \| --- \| |
| 2020#ASD | \| 0.04 \| 0.14 \| 0.29 \| -0.24 to 0.32 \| .770 \| \| --- \| --- \| --- \| --- \| --- \| |
| 2021#ADHD | \| 0.06 \| 0.26 \| 0.23 \| -0.45 to 0.57 \| .815 \| \| --- \| --- \| --- \| --- \| --- \| |
| 2021#ASD | \| -0.19 \| 0.21 \| -0.92 \| -0.60 to 0.22 \| .357 \| \| --- \| --- \| --- \| --- \| --- \| |
| Intercept (reference groups) | \| 0.00 \| 0.31 \| 0.00 \| -0.61 to 0.61 \| .999 \| \| --- \| --- \| --- \| --- \| --- \| |
|  |  |
|  | **Random effect** |
|  | \| Estimate \| SE \| 95% CI \| \| --- \| --- \| --- \| |
| Variance (intercept) | \| 0.51 \| 0.04 \| 0.45 to 0.59 \| \| --- \| --- \| --- \| |
| Variance (residual) | \| 0.38 \| 0.03 \| 0.32 to 0.45 \| \| --- \| --- \| --- \| |
| ICC | \| 0.58 \| 0.03 \| 0.52 to 0.63 \| \| --- \| --- \| --- \| |

ADHD: attention-deficit/hyperactivity disorder; ASD: autism spectrum disorder; ID: intellectual disability.

b: fixed effect coefficient; ICC: intraclass correlation coefficient; SE: standard error; z: z-score; 95% CI: 95% confidence interval; p: p-value; #: interaction term.

*Outcomes were z-standardised within each timepoint (pre-pandemic, 2020, 2021); therefore, coefficients for time reflect relative changes in symptoms.

Note: This table represents data from linear mixed modelling using the analytic sample, which included young people within the larger CAMHS (child and adolescent mental health services) cohort with complete sociodemographic and clinical characteristic data and any emotional and behavioural symptoms data in pre-pandemic and 2020 and/or 2021; Inverse probability weights were applied to the analysis, which were based on logistic regressions examining whether study predictors were associated with inclusion in the analytic sample (having emotional and behavioural symptoms data in pre-pandemic and 2020-2021). From these models, each participant’s predicted probability of inclusion was obtained, and inverse probability weights were derived as the reciprocal of these probabilities.

**Table S11.** Estimated trajectories of emotional symptoms between pre-pandemic and 2021 by primary diagnosis with inverse probability weights.

|  | **Fixed effect** | | | | | | | | | | | | | | |
| --- | --- | --- | --- | --- | --- | --- | --- | --- | --- | --- | --- | --- | --- | --- | --- |
|  | Emotional disorders (n=107) | | | | | ADHD (n=84) | | | | | ASD (n=197) | | | | |
| **Predictor** | b | SE | Z | 95% CI | p | b | SE | Z | 95% CI | p | b | SE | Z | 95% CI | p |
| Year (slope)* |  |  |  |  |  |  |  |  |  |  |  |  |  |  |  |
| Pre-pandemic | Reference | | | | | | | | | | | | | | |
| 2020 | 0.09 | 0.11 | 0.80 | -0.13 to 0.32 | .426 | -0.14 | 0.14 | -0.95 | -0.42 to 0.14 | .344 | -0.22 | 0.11 | -1.96 | -0.44 to -0.00 | .049 |
| 2021 | 0.12 | 0.14 | 0.87 | -0.16 to 0.40 | .383 | 0.22 | 0.18 | 1.19 | -0.14 to 0.58 | .232 | -0.36 | 0.14 | -2.47 | -0.64 to -0.07 | .013 |
| Age at start of pandemic | 0.02 | 0.03 | 0.83 | -0.03 to 0.08 | .409 | 0.02 | 0.03 | 0.76 | -0.03 to 0.07 | .447 | 0.01 | 0.02 | 0.60 | -0.03 to 0.06 | .549 |
| Sex |  |  |  |  |  |  |  |  |  |  |  |  |  |  |  |
| Male | Reference | | | | | | | | | | | | | | |
| Female | 0.22 | 0.16 | 1.37 | -0.10 to 0.54 | .170 | 0.08 | 0.20 | 0.39 | -0.31 to 0.47 | .694 | 0.22 | 0.12 | 1.89 | -0.01 to 0.45 | .059 |
| Ethnicity |  |  |  |  |  |  |  |  |  |  |  |  |  |  |  |
| White | Reference | | | | | | | | | | | | | | |
| Black | -0.05 | 0.19 | -0.25 | -0.43 to 0.33 | .804 | 0.05 | 0.22 | 0.24 | -0.38 to 0.49 | .808 | -0.12 | 0.19 | -0.63 | -0.48 to 0.25 | .531 |
| Asian, Mixed, or Other | 0.00 | 0.21 | 0.01 | -0.40 to 0.41 | .989 | -0.06 | 0.20 | -0.30 | -0.45 to 0.33 | .762 | -0.06 | 0.16 | -0.39 | -0.38 to 0.25 | .694 |
| Not stated | -0.13 | 0.19 | -0.71 | -0.50 to 0.24 | .475 | -0.37 | 0.29 | -1.28 | -0.94 to 0.20 | .201 | -0.27 | 0.15 | -1.76 | -0.57 to 0.03 | .078 |
| Neighbourhood deprivation |  |  |  |  |  |  |  |  |  |  |  |  |  |  |  |
| Not deprived | Reference | | | | | | | | | | | | | | |
| Deprived | 0.24 | 0.16 | 1.49 | -0.08 to 0.56 | .136 | 0.31 | 0.17 | 1.81 | -0.03 to 0.65 | .071 | 0.10 | 0.12 | 0.87 | -0.13 to 0.33 | .385 |
| Intellectual disability |  |  |  |  |  |  |  |  |  |  |  |  |  |  |  |
| No or not stated | Reference | | | | | | | | | | | | | | |
| Yes | Omitted | | | | | Omitted | | | | | -0.45 | 0.21 | -2.11 | -0.87 to -0.03 | .035 |
| Intercept (reference groups) | -0.53 | 0.43 | -1.23 | -1.36 to 0.31 | .217 | -0.56 | 0.33 | -1.72 | -1.20 to 0.08 | .085 | 0.22 | 0.25 | 0.88 | -0.27 to 0.71 | .380 |
|  |  |  |  |  |  |  |  |  |  |  |  |  |  |  |  |
|  | **Random effect** | | | | | | | | | | | | | | |
|  | Estimate | | SE | 95% CI | | Estimate | | SE | 95% CI | | Estimate | | SE | 95% CI | |
| Variance (intercept) | 0.52 | | 0.07 | 0.40 to 0.68 | | 0.54 | | 0.07 | 0.41 to 0.70 | | 0.51 | | 0.06 | 0.41 to 0.64 | |
| Variance (residual) | 0.32 | | 0.04 | 0.25 to 0.41 | | 0.30 | | 0.06 | 0.20 to 0.44 | | 0.40 | | 0.04 | 0.34 to 0.48 | |
| ICC | 0.62 | | 0.05 | 0.52 to 0.71 | | 0.64 | | 0.06 | 0.52 to 0.74 | | 0.56 | | 0.04 | 0.48 to 0.63 | |

ADHD: attention-deficit/hyperactivity disorder; ASD: autism spectrum disorder; ID: intellectual disability.

b: fixed effect coefficient; ICC: intraclass correlation coefficient; SE: standard error; z: z-score; 95% CI: 95% confidence interval; p: p-value; #: interaction term.

*Outcomes were z-standardised within each timepoint (pre-pandemic, 2020, 2021); therefore, coefficients for time reflect relative changes in symptoms.

Note: This table represents data from linear mixed modelling using the analytic sample, which included young people within the larger CAMHS (child and adolescent mental health services) cohort with complete sociodemographic and clinical characteristic data and any emotional and behavioural symptoms data in pre-pandemic and 2020 and/or 2021; Inverse probability weights were applied to the analysis, which were based on logistic regressions examining whether study predictors were associated with inclusion in the analytic sample (having emotional and behavioural symptoms data in pre-pandemic and 2020-2021). From these models, each participant’s predicted probability of inclusion was obtained, and inverse probability weights were derived as the reciprocal of these probabilities.

**Table S12.** Estimated trajectories of behavioural symptoms between pre-pandemic and 2021 by primary diagnosis with inverse probability weights.

|  | **Fixed effect** | | | | | | | | | | | | | | |
| --- | --- | --- | --- | --- | --- | --- | --- | --- | --- | --- | --- | --- | --- | --- | --- |
|  | Emotional disorders (n=107) | | | | | ADHD (n=84) | | | | | ASD (n=197) | | | | |
| **Predictor** | b | SE | Z | 95% CI | p | b | SE | Z | 95% CI | p | b | SE | Z | 95% CI | p |
| Year (slope)* |  |  |  |  |  |  |  |  |  |  |  |  |  |  |  |
| Pre-pandemic | Reference | | | | | | | | | | | | | | |
| 2020 | -0.07 | 0.10 | -0.71 | -0.27 to 0.12 | .479 | -0.12 | 0.22 | -0.53 | -0.55 to 0.32 | .597 | -0.00 | 0.09 | -0.05 | -0.19 to 0.18 | .959 |
| 2021 | -0.09 | 0.16 | -0.57 | -0.40 to 0.22 | .566 | 0.04 | 0.17 | 0.21 | -0.30 to 0.38 | .832 | -1.94 | 0.12 | -1.94 | -0.48 to -0.00 | .052 |
| Age at start of pandemic | -0.02 | 0.03 | -0.70 | -0.07 to 0.03 | .483 | 0.01 | 0.03 | 0.42 | -0.04 to 0.06 | .671 | -0.06 | 0.02 | -3.22 | -0.10 to -0.02 | .001 |
| Sex |  |  |  |  |  |  |  |  |  |  |  |  |  |  |  |
| Male | Reference | | | | | | | | | | | | | | |
| Female | -0.11 | 0.16 | -0.69 | -0.42 to 0.20 | .491 | -0.11 | 0.18 | -0.63 | -0.47 to 0.24 | .527 | 0.09 | 0.11 | 0.76 | -0.13 to 0.30 | .448 |
| Ethnicity |  |  |  |  |  |  |  |  |  |  |  |  |  |  |  |
| White | Reference | | | | | | | | | | | | | | |
| Black | -0.11 | 0.19 | -0.56 | -0.49 to 0.27 | .576 | 0.08 | 0.20 | 0.39 | -0.32 to 0.48 | .694 | 0.01 | 0.13 | 0.07 | -0.25 to 0.27 | .942 |
| Asian, Mixed, or Other | -0.13 | 0.21 | -0.59 | -0.55 to 0.29 | .557 | 0.27 | 0.22 | 1.25 | -0.15 to 0.69 | .212 | -0.01 | 0.16 | -0.07 | -0.32 to 0.30 | .945 |
| Not stated | -0.32 | 0.25 | -1.30 | -0.81 to 0.16 | .194 | 0.44 | 0.18 | 2.46 | 0.09 to 0.79 | .014 | -0.19 | 0.13 | -1.45 | -0.44 to 0.07 | .146 |
| Neighbourhood deprivation |  |  |  |  |  |  |  |  |  |  |  |  |  |  |  |
| Not deprived | Reference | | | | | | | | | | | | | | |
| Deprived | 0.10 | 0.27 | 0.35 | -0.44 to 0.63 | .725 | 0.10 | 0.20 | 0.52 | -0.29 to 0.50 | .603 | 0.16 | 0.12 | 1.35 | -0.07 to 0.39 | .177 |
| Intellectual disability |  |  |  |  |  |  |  |  |  |  |  |  |  |  |  |
| No or not stated | Reference | | | | | | | | | | | | | | |
| Yes | Omitted | | | | | Omitted | | | | | 0.10 | 0.18 | 0.57 | -0.25 to 0.45 | .569 |
| Intercept (reference groups) | 0.11 | 0.40 | 0.29 | -0.66 to 0.89 | .774 | -0.03 | 0.31 | -0.08 | -0.63 to 0.58 | .932 | 0.89 | 0.23 | 3.91 | 0.44 to 1.34 | .000 |
|  |  |  |  |  |  |  |  |  |  |  |  |  |  |  |  |
|  | **Random effect** | | | | | | | | | | | | | | |
|  | Estimate | | SE | 95% CI | | Estimate | | SE | 95% CI | | Estimate | | SE | 95% CI | |
| Variance (intercept) | 0.61 | | 0.08 | 0.47 to 0.78 | | 0.45 | | 0.08 | 0.31 to 0.65 | | 0.46 | | 0.05 | 0.37 to 0.57 | |
| Variance (residual) | 0.28 | | 0.04 | 0.21 to 0.37 | | 0.51 | | 0.07 | 0.38 to 0.67 | | 0.34 | | 0.03 | 0.28 to 0.40 | |
| ICC | 0.69 | | 0.05 | 0.59 to 0.77 | | 0.47 | | 0.06 | 0.35 to 0.59 | | 0.58 | | 0.04 | 0.50 to 0.65 | |

ADHD: attention-deficit/hyperactivity disorder; ASD: autism spectrum disorder; ID: intellectual disability.

b: fixed effect coefficient; ICC: intraclass correlation coefficient; SE: standard error; z: z-score; 95% CI: 95% confidence interval; p: p-value; #: interaction term.

*Outcomes were z-standardised within each timepoint (pre-pandemic, 2020, 2021); therefore, coefficients for time reflect relative changes in symptoms.

Note: This table represents data from linear mixed modelling using the analytic sample, which included young people within the larger CAMHS (child and adolescent mental health services) cohort with complete sociodemographic and clinical characteristic data and any emotional and behavioural symptoms data in pre-pandemic and 2020 and/or 2021; Inverse probability weights were applied to the analysis, which were based on logistic regressions examining whether study predictors were associated with inclusion in the analytic sample (having emotional and behavioural symptoms data in pre-pandemic and 2020-2021). From these models, each participant’s predicted probability of inclusion was obtained, and inverse probability weights were derived as the reciprocal of these probabilities.

**Table S13.** Estimated trajectories of emotional symptoms between pre-pandemic and 2021 without weights.

|  | **Fixed effect** |
| --- | --- |
| **Predictor** | \| b \| SE \| Z \| 95% CI \| p \| \| --- \| --- \| --- \| --- \| --- \| |
| Year (slope)* |  |
| Pre-pandemic | Reference |
| 2020 | \| 0.08 \| 0.08 \| 1.01 \| -0.08 to 0.24 \| .312 \| \| --- \| --- \| --- \| --- \| --- \| |
| 2021 | \| -0.00 \| 0.10 \| -0.04 \| -0.19 to 0.19 \| .971 \| \| --- \| --- \| --- \| --- \| --- \| |
| Age at start of pandemic | \| 0.02 \| 0.01 \| 1.53 \| -0.01 to 0.04 \| .126 \| \| --- \| --- \| --- \| --- \| --- \| |
| Sex |  |
| Male | Reference |
| Female | \| 0.19 \| 0.08 \| 2.32 \| 0.03 to 0.35 \| .020 \| \| --- \| --- \| --- \| --- \| --- \| |
| Ethnicity |  |
| White | Reference |
| Black | \| 0.10 \| 0.14 \| 0.75 \| -0.17 to 0.38 \| .455 \| \| --- \| --- \| --- \| --- \| --- \| |
| Asian, Mixed, or Other | \| -0.04 \| 0.14 \| -0.27 \| -0.30 to 0.23 \| .790 \| \| --- \| --- \| --- \| --- \| --- \| |
| Not stated | \| -0.12 \| 0.13 \| -0.91 \| -0.37 to 0.14 \| .364 \| \| --- \| --- \| --- \| --- \| --- \| |
| Neighbourhood deprivation |  |
| Not deprived | Reference |
| Deprived | \| 0.20 \| 0.10 \| 2.02 \| 0.01 to 0.38 \| .043 \| \| --- \| --- \| --- \| --- \| --- \| |
| Primary diagnosis |  |
| Emotional disorders | Reference |
| ADHD | \| -0.18 \| 0.12 \| -1.58 \| -0.41 to 0.04 \| .114 \| \| --- \| --- \| --- \| --- \| --- \| |
| ASD | \| 0.33 \| 0.10 \| 3.42 \| 0.14 to 0.52 \| .001 \| \| --- \| --- \| --- \| --- \| --- \| |
| Intellectual disability |  |
| No or not stated | Reference |
| Yes | \| -0.45 \| 0.26 \| -1.72 \| -0.96 to 0.06 \| .085 \| \| --- \| --- \| --- \| --- \| --- \| |
| Year#Ethnicity |  |
| 2020#Black | \| -0.43 \| 0.17 \| -2.57 \| -0.76 to -0.10 \| .010 \| \| --- \| --- \| --- \| --- \| --- \| |
| 2020#Asian, mixed and other | \| -0.14 \| 0.17 \| -0.85 \| -0.47 to 0.19 \| .393 \| \| --- \| --- \| --- \| --- \| --- \| |
| 2020#Not stated | \| -0.30 \| 0.16 \| -1.92 \| -0.61 to 0.01 \| .055 \| \| --- \| --- \| --- \| --- \| --- \| |
| 2021#Black | \| -0.03 \| 0.22 \| -0.16 \| -0.46 to 0.39 \| .876 \| \| --- \| --- \| --- \| --- \| --- \| |
| 2021#Asian, mixed and other | \| 0.21 \| 0.19 \| 1.07 \| -0.17 to 0.59 \| .282 \| \| --- \| --- \| --- \| --- \| --- \| |
| 2021#Not stated | \| -0.15 \| 0.19 \| -0.79 \| -0.51 to 0.22 \| .429 \| \| --- \| --- \| --- \| --- \| --- \| |
| Intercept (reference groups) | \| -0.39 \| 0.20 \| -1.99 \| -0.78 to -0.01 \| .047 \| \| --- \| --- \| --- \| --- \| --- \| |
|  |  |
|  | **Random effect** |
|  | \| Estimate \| SE \| 95% CI \| \| --- \| --- \| --- \| |
| Variance (intercept) | \| 0.30 \| 0.04 \| 0.22 to 0.39 \| \| --- \| --- \| --- \| |
| Variance (residual) | \| 0.57 \| 0.04 \| 0.50 to 0.64 \| \| --- \| --- \| --- \| |
| ICC | \| 0.34 \| 0.04 \| 0.27 to 0.42 \| \| --- \| --- \| --- \| |

ADHD: attention-deficit/hyperactivity disorder; ASD: autism spectrum disorder; ID: intellectual disability.

b: fixed effect coefficient; ICC: intraclass correlation coefficient; SE: standard error; z: z-score; 95% CI: 95% confidence interval; p: p-value; #: interaction term.

*Outcomes were z-standardised within each timepoint (pre-pandemic, 2020, 2021); therefore, coefficients for time reflect relative changes in symptoms.

Note: This table represents data from linear mixed modelling using the analytic sample, which included young people within the larger CAMHS (child and adolescent mental health services) cohort with complete sociodemographic and clinical characteristic data and any emotional and behavioural symptoms data in pre-pandemic and 2020 and/or 2021.

**Table S14.** Estimated trajectories of emotional symptoms between pre-pandemic and 2021 by primary diagnosis without weights.

|  | **Fixed effect** | | | | | | | | | | | | | | |
| --- | --- | --- | --- | --- | --- | --- | --- | --- | --- | --- | --- | --- | --- | --- | --- |
|  | Emotional disorders (n=107) | | | | | ADHD (n=84) | | | | | ASD (n=197) | | | | |
| **Predictor** | B | SE | Z | 95% CI | p | B | SE | Z | 95% CI | p | b | SE | Z | 95% CI | p |
| Year (slope)* |  |  |  |  |  |  |  |  |  |  |  |  |  |  |  |
| Pre-pandemic | Reference | | | | | | | | | | | | | | |
| 2020 | 0.03 | 0.11 | 0.27 | -0.19 to 0.25 | .791 | -0.08 | 0.11 | -0.74 | -0.31 to 0.14 | .458 | -0.10 | 0.08 | -1.21 | -0.27 to 0.06 | .225 |
| 2021 | 0.11 | 0.14 | 0.80 | -0.16 to 0.39 | .424 | 0.16 | 0.13 | 1.24 | -0.10 to 0.42 | .216 | -0.13 | 0.10 | -1.36 | -0.33 to 0.06 | .175 |
| Age at start of pandemic | 0.02 | 0.02 | 1.00 | -0.02 to 0.07 | .320 | 0.02 | 0.03 | 0.76 | -0.03 to 0.07 | .446 | 0.01 | 0.02 | 0.73 | -0.02 to 0.05 | .467 |
| Sex |  |  |  |  |  |  |  |  |  |  |  |  |  |  |  |
| Male | Reference | | | | | | | | | | | | | | |
| Female | 0.20 | 0.15 | 1.32 | -0.10 to 0.49 | .188 | 0.07 | 0.19 | 0.37 | -0.31 to 0.45 | .709 | 0.23 | 0.12 | 2.02 | 0.01 to 0.46 | .043 |
| Ethnicity |  |  |  |  |  |  |  |  |  |  |  |  |  |  |  |
| White | Reference | | | | | | | | | | | | | | |
| Black | -0.05 | 0.19 | -0.23 | -0.43 to 0.34 | .815 | 0.01 | 0.21 | 0.06 | -0.41 to 0.43 | .954 | -0.10 | 0.18 | -0.58 | -0.46 to 0.25 | .564 |
| Asian, Mixed, or Other | 0.00 | 0.21 | 0.02 | -0.40 to 0.41 | .983 | -0.05 | 0.22 | -0.23 | -0.49 to 0.39 | .819 | -0.05 | 0.16 | -0.32 | -0.36 to 0.26 | .750 |
| Not stated | -0.13 | 0.21 | -0.62 | -0.53 to 0.28 | .533 | -0.38 | 0.25 | -1.53 | -0.87 to 0.11 | .125 | -0.27 | 0.14 | -1.95 | -0.54 to 0.00 | .051 |
| Neighbourhood deprivation |  |  |  |  |  |  |  |  |  |  |  |  |  |  |  |
| Not deprived | Reference | | | | | | | | | | | | | | |
| Deprived | 0.26 | 0.24 | 1.05 | -0.22 to 0.73 | .292 | 0.33 | 0.21 | 1.59 | -0.08 to 0.74 | .111 | 0.11 | 0.12 | 0.90 | -0.13 to 0.35 | .371 |
| Intellectual disability |  |  |  |  |  |  |  |  |  |  |  |  |  |  |  |
| No or not stated | Reference | | | | | | | | | | | | | | |
| Yes | Omitted | | | | | Omitted | | | | | -0.45 | 0.26 | -1.70 | -0.96 to 0.07 | .089 |
| Intercept (reference groups) | -0.49 | 0.35 | -1.40 | -1.19 to 0.20 | .163 | -0.56 | 0.34 | -1.66 | -1.23 to 0.10 | .096 | 0.12 | 0.23 | 0.52 | -0.33 to 0.57 | .603 |
|  |  |  |  |  |  |  |  |  |  |  |  |  |  |  |  |
|  | **Random effect** | | | | | | | | | | | | | | |
|  | Estimate | | SE | 95% CI | | Estimate | | SE | 95% CI | | Estimate | | SE | 95% CI | |
| Variance (intercept) | 0.28 | | 0.08 | 0.16 to 0.49 | | 0.35 | | 0.09 | 0.21 to 0.57 | | 0.27 | | 0.06 | 0.18 to 0.42 | |
| Variance (residual) | 0.59 | | 0.07 | 0.47 to 0.75 | | 0.46 | | 0.06 | 0.36 to 0.60 | | 0.61 | | 0.05 | 0.52 to 0.73 | |
| ICC | 0.32 | | 0.08 | 0.19 to 0.48 | | 0.43 | | 0.08 | 0.29 to 0.58 | | 0.31 | | 0.06 | 0.21 to 0.43 | |

ADHD: attention-deficit/hyperactivity disorder; ASD: autism spectrum disorder; ID: intellectual disability.

b: fixed effect coefficient; ICC: intraclass correlation coefficient; SE: standard error; z: z-score; 95% CI: 95% confidence interval; p: p-value; #: interaction term.

*Outcomes were z-standardised within each timepoint (pre-pandemic, 2020, 2021); therefore, coefficients for time reflect relative changes in symptoms.

Note: This table represents data from linear mixed modelling using the analytic sample, which included young people within the larger CAMHS (child and adolescent mental health services) cohort with complete sociodemographic and clinical characteristic data and any emotional and behavioural symptoms data in pre-pandemic and 2020 and/or 2021.

**Table S15.** Estimated trajectories of behavioural symptoms between pre-pandemic and 2021 without weights.

|  | **Fixed effect** |
| --- | --- |
| **Predictor** | \| b \| SE \| Z \| 95% CI \| p \| \| --- \| --- \| --- \| --- \| --- \| |
| Year (slope)* |  |
| Pre-pandemic | Reference |
| 2020 | \| 0.02 \| 0.06 \| 0.36 \| -0.10 to 0.14 \| .715 \| \| --- \| --- \| --- \| --- \| --- \| |
| 2021 | \| -0.06 \| 0.07 \| -0.86 \| -0.20 to 0.08 \| .388 \| \| --- \| --- \| --- \| --- \| --- \| |
| Age at start of pandemic | \| -0.04 \| 0.01 \| -2.87 \| -0.06 to -0.01 \| .004 \| \| --- \| --- \| --- \| --- \| --- \| |
| Sex |  |
| Male | Reference |
| Female | \| -0.00 \| 0.08 \| -0.02 \| -0.16 to 0.16 \| .982 \| \| --- \| --- \| --- \| --- \| --- \| |
| Ethnicity |  |
| White | Reference |
| Black | \| -0.05 \| 0.11 \| -0.47 \| -0.27 to 0.17 \| .640 \| \| --- \| --- \| --- \| --- \| --- \| |
| Asian, Mixed, or Other | \| 0.00 \| 0.11 \| 0.02 \| -0.21 to 0.21 \| .983 \| \| --- \| --- \| --- \| --- \| --- \| |
| Not stated | \| -0.11 \| 0.10 \| -1.11 \| -0.32 to 0.09 \| .266 \| \| --- \| --- \| --- \| --- \| --- \| |
| Neighbourhood deprivation |  |
| Not deprived | Reference |
| Deprived | \| 0.13 \| 0.10 \| 1.35 \| -0.06 to 0.32 \| .176 \| \| --- \| --- \| --- \| --- \| --- \| |
| Primary diagnosis |  |
| Emotional disorders | Reference |
| ADHD | \| 0.42 \| 0.12 \| 3.64 \| 0.19 to 0.65 \| .000 \| \| --- \| --- \| --- \| --- \| --- \| |
| ASD | \| 0.39 \| 0.10 \| 4.06 \| 0.20 to 0.58 \| .000 \| \| --- \| --- \| --- \| --- \| --- \| |
| Intellectual disability |  |
| No or not stated | Reference |
| Yes | \| -0.00 \| 0.26 \| -0.02 \| -0.51 to 0.50 \| .986 \| \| --- \| --- \| --- \| --- \| --- \| |
| Intercept (reference groups) | \| 0.19 \| 0.19 \| 0.95 \| -0.20 to 0.57 \| .341 \| \| --- \| --- \| --- \| --- \| --- \| |
|  |  |
|  | **Random effect** |
|  | \| Estimate \| SE \| 95% CI \| \| --- \| --- \| --- \| |
| Variance (intercept) | \| 0.27 \| 0.04 \| 0.20 to 0.36 \| \| --- \| --- \| --- \| |
| Variance (residual) | \| 0.62 \| 0.04 \| 0.55 to 0.70 \| \| --- \| --- \| --- \| |
| ICC | \| 0.30 \| 0.04 \| 0.23 to 0.38 \| \| --- \| --- \| --- \| |

ADHD: attention-deficit/hyperactivity disorder; ASD: autism spectrum disorder; ID: intellectual disability.

b: fixed effect coefficient; ICC: intraclass correlation coefficient; SE: standard error; z: z-score; 95% CI: 95% confidence interval; p: p-value; #: interaction term.

*Outcomes were z-standardised within each timepoint (pre-pandemic, 2020, 2021); therefore, coefficients for time reflect relative changes in symptoms.

Note: This table represents data from linear mixed modelling using the analytic sample, which included young people within the larger CAMHS (child and adolescent mental health services) cohort with complete sociodemographic and clinical characteristic data and any emotional and behavioural symptoms data in pre-pandemic and 2020 and/or 2021.

**Table S16.** Estimated trajectories of behavioural symptoms between pre-pandemic and 2021 by primary diagnosis without weights.

|  | **Fixed effect** | | | | | | | | | | | | | | |
| --- | --- | --- | --- | --- | --- | --- | --- | --- | --- | --- | --- | --- | --- | --- | --- |
|  | Emotional disorders (n=107) | | | | | ADHD (n=84) | | | | | ASD (n=197) | | | | |
| **Predictor** | b | SE | Z | 95% CI | p | b | SE | Z | 95% CI | p | b | SE | Z | 95% CI | p |
| Year (slope)* |  |  |  |  |  |  |  |  |  |  |  |  |  |  |  |
| Pre-pandemic | Reference | | | | | | | | | | | | | | |
| 2020 | -0.05 | 0.10 | -0.51 | -0.26 to 0.15 | .610 | -0.03 | 0.15 | -0.18 | -0.31 to 0.26 | .861 | 0.09 | 0.08 | 1.04 | -0.08 to 0.25 | .297 |
| 2021 | -0.06 | 0.13 | -0.49 | -0.32 to 0.19 | .625 | 0.01 | 0.17 | 0.04 | -0.32 to 0.34 | .967 | -0.11 | 0.10 | -1.16 | -0.31 to 0.08 | .245 |
| Age at start of pandemic | -0.02 | 0.03 | -0.86 | -0.07 to 0.03 | .392 | 0.01 | 0.02 | 0.59 | -0.03 to 0.06 | .554 | -0.06 | 0.02 | -3.58 | -0.10 to -0.03 | .000 |
| Sex |  |  |  |  |  |  |  |  |  |  |  |  |  |  |  |
| Male | Reference | | | | | | | | | | | | | | |
| Female | -0.11 | 0.16 | -0.65 | -0.42 to 0.21 | .518 | -0.11 | 0.17 | -0.65 | -0.45 to 0.22 | .516 | 0.09 | 0.11 | 0.85 | -0.12 to 0.31 | .394 |
| Ethnicity |  |  |  |  |  |  |  |  |  |  |  |  |  |  |  |
| White | Reference | | | | | | | | | | | | | | |
| Black | -0.10 | 0.21 | -0.50 | -0.51 to 0.31 | .620 | 0.02 | 0.19 | 0.08 | -0.36 to 0.39 | .935 | -0.00 | 0.17 | -0.01 | -0.34 to 0.34 | .992 |
| Asian, Mixed, or Other | -0.11 | 0.22 | -0.50 | -0.55 to 0.33 | .615 | 0.28 | 0.20 | 1.37 | -0.12 to 0.67 | .171 | -0.02 | 0.15 | -0.11 | -0.31 to 0.28 | .909 |
| Not stated | -0.31 | 0.22 | -1.37 | -0.74 to 0.13 | .171 | 0.45 | 0.22 | 2.06 | 0.02 to 0.88 | .039 | -0.20 | 0.13 | -1.53 | -0.46 to 0.06 | .126 |
| Neighbourhood deprivation |  |  |  |  |  |  |  |  |  |  |  |  |  |  |  |
| Not deprived | Reference | | | | | | | | | | | | | | |
| Deprived | 0.09 | 0.26 | 0.36 | -0.42 to 0.60 | .721 | 0.14 | 0.18 | 0.78 | -0.22 to 0.50 | .438 | 0.15 | 0.12 | 1.25 | -0.08 to 0.38 | .211 |
| Intellectual disability |  |  |  |  |  |  |  |  |  |  |  |  |  |  |  |
| No or not stated | Reference | | | | | | | | | | | | | | |
| Yes | Omitted | | | | | Omitted | | | | | 0.09 | 0.25 | 0.37 | -0.40 to 0.59 | .709 |
| Intercept (reference groups) | 0.15 | 0.38 | 0.38 | -0.60 to 0.89 | .704 | -0.08 | 0.31 | -0.28 | -0.69 to 0.52 | .783 | 0.86 | 0.22 | 3.89 | 0.43 to 1.29 | .000 |
|  |  |  |  |  |  |  |  |  |  |  |  |  |  |  |  |
|  | **Random effect** | | | | | | | | | | | | | | |
|  | Estimate | | SE | 95% CI | | Estimate | | SE | 95% CI | | Estimate | | SE | 95% CI | |
| Variance (intercept) | 0.41 | | 0.09 | 0.26 to 0.63 | | 0.10 | | 0.08 | 0.02 to 0.53 | | 0.23 | | 0.05 | 0.14 to 0.36 | |
| Variance (residual) | 0.50 | | 0.06 | 0.39 to 0.63 | | 0.79 | | 0.11 | 0.61 to 1.03 | | 0.61 | | 0.05 | 0.51 to 0.72 | |
| ICC | 0.45 | | 0.07 | 0.32 to 0.59 | | 0.11 | | 0.09 | 0.02 to 0.44 | | 0.27 | | 0.06 | 0.18 to 0.39 | |

ADHD: attention-deficit/hyperactivity disorder; ASD: autism spectrum disorder; ID: intellectual disability.

b: fixed effect coefficient; ICC: intraclass correlation coefficient; SE: standard error; z: z-score; 95% CI: 95% confidence interval; p: p-value; #: interaction term.

*Outcomes were z-standardised within each timepoint (pre-pandemic, 2020, 2021); therefore, coefficients for time reflect relative changes in symptoms.

Note: This table represents data from linear mixed modelling using the analytic sample, which included young people within the larger CAMHS (child and adolescent mental health services) cohort with complete sociodemographic and clinical characteristic data and any emotional and behavioural symptoms data in pre-pandemic and 2020 and/or 2021.
